# Salutogenic Environmental Health Model—proposing an integrative and interdisciplinary lens on the genesis of health

**DOI:** 10.3389/fpubh.2024.1445181

**Published:** 2024-10-17

**Authors:** Jule Anna Pleyer, Laura Dominique Pesliak, Annette Konstanze Fides Malsch, Timothy McCall

**Affiliations:** ^1^Department of Environment and Health, School of Public Health, Bielefeld University, Bielefeld, Germany; ^2^Joint Institute for Individualisation in a Changing Environment, University, Münster and Bielefeld, Germany; ^3^Department of Sustainable Environmental Health Sciences, Medical School OWL, Bielefeld University, Bielefeld, Germany

**Keywords:** salutogenesis, environment, health model, Antonovsky, interdisciplinary, theoretical analysis, determinants, health promotion

## Abstract

**Introduction:**

The maintenance of health is a central objective of public health initiatives. Within the salutogenic paradigm, health promotion is focused on understanding the mechanisms of health development. Models serve as indispensable tools. One of the leading paradigms in the health sciences is the Salutogenic Model developed by Aaron Antonovsky. However, it lacks sufficient specification to reflect the complexity of the environmental dimensions that have emerged from research in environmental health science. The interactions and impact pathways between these dimensions on health status are not adequately distinguished. The objective of this study is to address this gap by extending Antonovsky’s model to encompass environmental dimensions, that is, the interactions between humans and their environment. Furthermore, the study will integrate examples of models and theories from various disciplines to illustrate how a more comprehensive and holistic explanation of health development can be provided from an interdisciplinary environmental public health perspective.

**Methodology:**

As part of a three-step approach, a Basic Model was first developed that integrates the five environmental dimensions—Natural, Built-Material, Socio-Cultural, Psycho-Social, and Individual—into the Salutogenic Model. Subsequently, narrative non-exhaustive literature research was used to identify interdisciplinary example models. The models were used to identify gaps in the Basic Model through a critical lens and to synthesize them into a more holistic model.

**Results:**

The synthesis of fifteen interdisciplinary models resulted in the development of an integrative Salutogenic Environmental Health Model (SEHM), which comprises twelve principal components of health development and their interactions and pathways. Links to the original models permit the user to refer back to them.

**Discussion:**

This integrative approach offers a comprehensive understanding of the development of health by synthesizing disparate explanatory models and theories from various disciplines through theoretical analysis. The various environmental dimensions and the determinants of health contained therein, as well as their mental and physical processing and the associated components of health development and their interactions, are summarized in this new model. The SEHM thus enables a differentiated analysis of health determinants and serves as an operational framework for health promotion and pathways to well-being in contemporary research contexts.

## 1 Introduction

The preservation of health is a crucial human asset and constitutes the primary goal of public health endeavors (1). Defined by the World Health Organization (WHO) as the “science and art of promoting health, preventing disease, and prolonging life through organized societal efforts” (2) (p. 3), health promotion empowers individuals to assert greater control over their well-being by influencing pertinent determinants, thus contributing to the comprehensive analysis and fortification of health resources across all societal strata (3, 4). Within this salutogenic paradigm, health promotion systematically addresses the mechanisms and locations where health is generated (4).

Human health and well-being are highly dependent on the quality of the environment (5). Forming a sub-discipline of public health, the scientific discipline of environmental public health, as defined by the American Public Health Association (6), is concerned with analyzing the relationship between health and the environment. Additionally, as outlined by the German Robert-Koch-Institut (7), it encompasses the influence of the environment on human health at the population level (5).

The elucidation of the dynamics underpinning the genesis of health and illness mandates a comprehensive approach, wherein various environmental dimensions, characterized by their interdisciplinary nature (8), assume prominence as health determinants. From the perspective of environmental public health, these determinants must be considered within the contextual framework of both health conditions and individual health behavior (9, 10).

In this context, models serve as indispensable theoretical instruments in (environmental) public health research and emerge as essential tools for unraveling the complexities of health development. They also play a vital role in enhancing healthcare systems and comprehending the complex interplay between health and influential factors (11).

### 1.1 Propelling theoretical progress: shortcomings of Antonovsky’s Salutogenic Model

Embedded within this discourse on health dynamics, Antonovsky’s Salutogenic Model (1979) stands as a pivotal contribution and a leading paradigm within the health sciences. Distinct from the prevailing pathogenic orientation, this model advocates for a nuanced exploration into the origins of health and the intrinsic capacity for well-being, steering away from a solely pathogenic approach. Antonovsky’s model (1979) can hence be construed as a foundational concept in the realm of health promotion (12). In contrast to the dichotomous understanding of health and illness, Antonovsky (13, 14) developed a salutogenic view that considers health as a multifaceted state or condition of the human organism on a multidimensional continuum between ease and disease. Health is the result of a long chain of phenomena and moves along the continuum in time and social space, depending on various factors and pathways.

**Figure 1:**
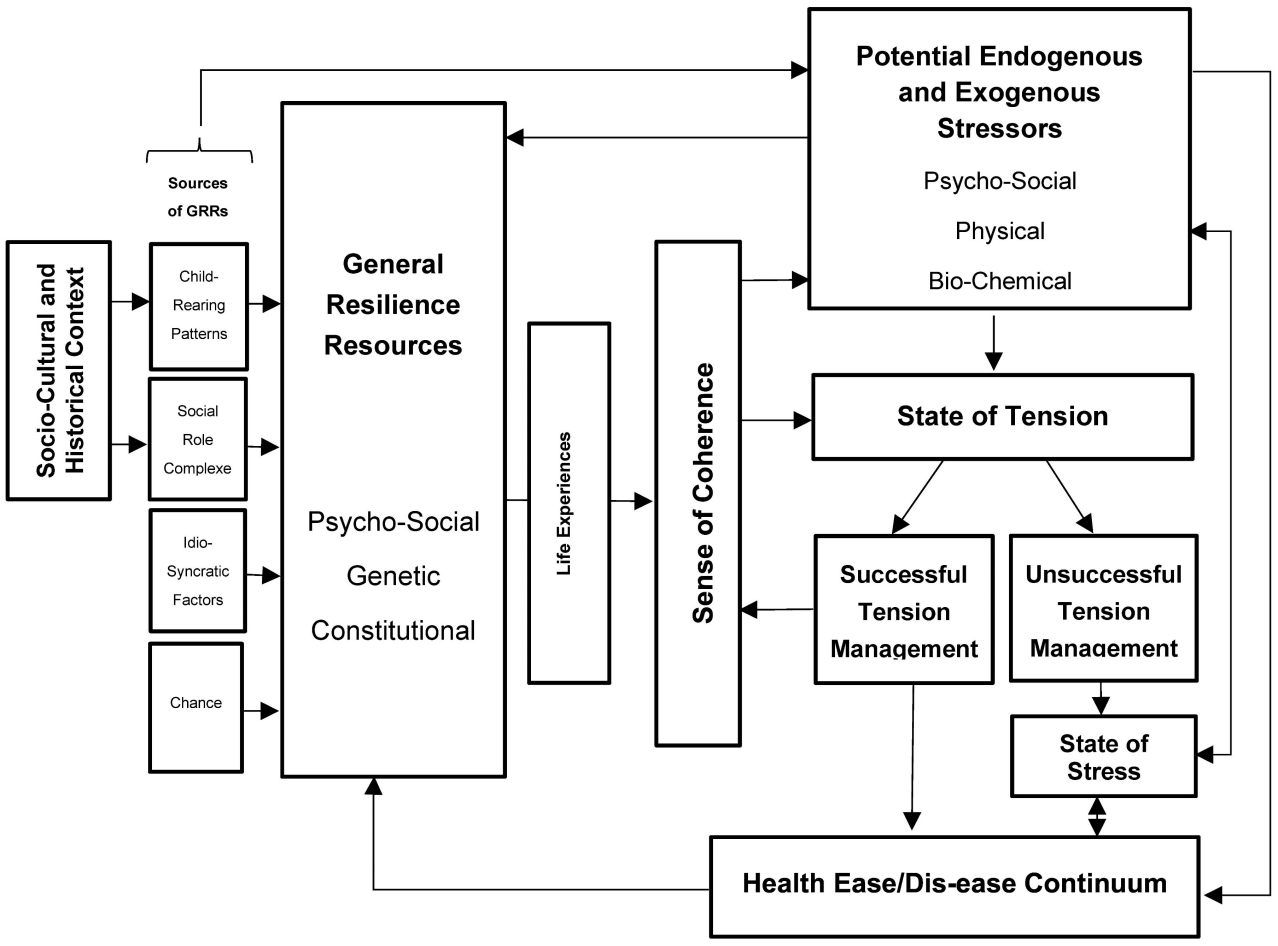
Own figure of the Salutogenic Model based on Antonovsky (14), p. 184ff.

Figure 1 demonstrates how Antonovsky’s theory places a special emphasis on the *Sense of Coherence* as the core element in explaining the movement on the *Ease/Dis-ease Continuum* [as coined by Antonovsky (14)]. This *Sense of Coherence* forms an essential component of a person’s personality structure and represents a “*global orientation that expresses the extent to which one has a pervasive, enduring, though dynamic feeling of confidence that one’s internal and external environments are predictable and that there is a high probability that things will work out as well as can reasonably be expected*” [(14), p. 184]. Individuals with a strong *Sense of Coherence (SoC)* are able to see reality and have a strong belief that things will turn out well for them, whereas individuals with a weak *SoC* are more likely to assume a negative outcome. It is important to note that one’s *SoC* is not predetermined by genetics or early childhood experiences, but rather can be assessed, developed, reinforced, and altered throughout one’s lifetime (14).

According to the Salutogenic Model, the extent to which *Life Experience* (including child-rearing patterns, social role complexes, idiosyncratic factors, and chances in life) provides people with *Generalized Resilience Resources (GRRs; Resources),* determines the development of a strong *SoC*. *GRRs* are “*characteristics of the person, the group, or the environment that can facilitate effective tension management*” [(14), p. 99]. This implies dealing with a *State of Tension* that is triggered by confrontation with *Stressors* and can have pathological, neutral, or beneficial consequences. *Stressors*, which can be any given phenomenon, experience, or stimulus, only lead to a shift on the continuum toward health ease or *dis-ease* [as coined by Antonovsky (14)], depending on individual assessment and corresponding *Tension Management*. *Successful Tension Management* can be achieved by someone with a strong *SoC* through the use of *GRRs,* or by preventing the transformation of tension into *Stress*. *Successful Tension Management* can be regarded as a positive *Life Experience* that contributes to the development of a strong *SoC* and a shift of the individual’s location on the *Ease/Dis-ease Continuum* toward the ease end, whereas *Unsuccessful Tension Management* can lead to *Stress*, contributing to a shift toward the *Dis-ease* end of the continuum (14).

The fundamental assumption of the Salutogenic Model has been corroborated through extensive research (15–17). However, the model represents merely an initial attempt at formulating an explanation for the genesis of health, necessitating expansion in light of new insights from the current state of research. While the model was a pioneering effort in the context of the research landscape at the time and has remained valuable for a fundamental understanding of salutogenesis, it has nevertheless become outdated in light of advances in research and the growing complexity of human-environment interactions in the Anthropocene. In this regard, the Salutogenic Model lacks specification of the defined health determinants’ complexity. These determinants emerge from the individual and environmental circumstances of people, exerting influence on health, yet they are neither precisely delineated nor embedded in the context of the various environmental dimensions that provide the framework for explaining the origins of health and illness. The Health Map by Barton and Grant (18) can be employed to elucidate the issue in greater detail. It is recognized as the most important environmental health framework structuring the different environmental dimensions shaping human health. The *Natural Environment* is defined as encompassing nature and its bio-physical properties, as distinct from the *Built-Material Environment*, which consists exclusively of human-made, purposeful spaces and structures, along with the connecting mobility and infrastructural systems within which human activities occur (18). Non-physical, anthropogenic dimensions emerge within the *Socio-Cultural Environment*, implying social, economic, political, and legal structures, such as the working environment, social networks, and communities (18). Finally, the dimension of the *Individual* is delineated, referring to demographic variables, genetic conditions, vulnerability, and physiological needs (18).

The Health Map demonstrates that the *Stressors* and *GRRs* addressed by Antonovsky require a more nuanced differentiation with regard to the diversity of these environmental dimensions in order to be able to explain the genesis of health in a nuanced way from an environmental perspective. An environment-related health model should incorporate the current state of knowledge about the determinants of the different environmental dimensions and their pathways to the genesis of health. In Antonovky’s model (1979), endogenous and exogenous *Stressors* are explicitly characterized as bio-chemically, physically, and psycho-socially instantiated. This compromises the comprehensive appreciation of the multidimensional spectrum of stressors resulting from the Health Map (see Table 1). The consideration of natural stressors in this framework is particularly insufficient. In this context, the model lacks a comprehensive explanatory framework to explicate the genesis of health (or disease) in response to specific natural stressors. Consequently, from a holistic (environmental) public health perspective, the model is inadequate. Similar criticism applies to the *Generalized Resistance Resources*. According to Antonovsky (14), they represent healing resistance potentials that keep potentially pathogenic factors away from people from the outset and contribute to coping with *States of Tension*, thereby strengthening the SoC (14, 19). Antonovsky’s model (1979) categorizes mainly psycho-social, genetic, and constitutional factors as *GRRs*. The role of the natural environment and its bio-physical properties as a health resource remains unrecognized (see Table 1). This represents a significant deficiency from both a salutogenic and pathogenic perspective, given that the natural environment and interactions with it constitute essential resources for health (20–22), while exposure to environmental stressors, such as air pollution or the impacts of climate change, ranks among the most current global health threats (23). This dynamic potential of environmental determinants, denoted in this paper as the “continuum potential,” and the implicit acknowledgement of such dynamic properties of health determinants as either resources or stressors in diverse contexts is absent in Antonovsky’s model.

**Table 1:** Gaps in the Salutogenic Model and the Health Map in relation to the environmental dimensions.

	Salutogenesis Model (14)		Health Map (18)
Environmental dimensions	Stressors	Resources	Determinants
Individual	✓	✓	✓
Psycho-Social	✓	✓	×
Social-Cultural	×	✓	✓
Built-Material	✓	×	✓
Natural	×	×	✓

*GRRs* are also understood as assets that contribute to a strong *SoC* through the formation of *Positive Life Experiences* that promote *Successful Tension Management*. However, the model undermines the human-environment interaction and its resulting outcomes by failing to consider the interplay between natural environments, life circumstances, and individual characteristics. The absence of an integrative environmental perspective undermines inequalities in health opportunities, stressor distribution, and resource allocation, as these arise from the interplay of environmental conditions and human-environment interactions (24, 25). This impairs the ability of the model to serve as an explanatory framework for health development, particularly in the context of a salutogenic environmental model.

“To summarize, although Antonovsky’s model is important in demonstrating the general mechanisms of health and disease in his time, the state of knowledge about health determinants in different environmental dimensions and their pathways has expanded enormously in many different disciplines since then. Accordingly, Antonovsky’s model does not adequately capture the complexity of health determinants and their continuum potential within the various environmental spheres (see Table 1). This deficiency results in an insufficient emphasis on human-environment interaction and necessitates a theoretical augmentation of the salutogenic paradigm to enhance our understanding of health development from an environmental public health perspective. Therefore, we propose that a more detailed refinement of the model can enhance its applicability for contemporary and future research.”

### 1.2 The role of the human-environment interaction in augmenting the Salutogenic Model

The public health relevance of human-environment interaction was initially emphasized from a salutogenic perspective as a crucial aspect of physical, mental, and social well-being by the Ottawa Charter for Health Promotion (26). This emphasis forms the basis for a social-ecological pathway of health (26, 27). Based on this interplay between humans and the environment, health promotion incorporates the protection of natural and social resources into its concept. Through the core strategy of the setting approach (28, 29), it directs attention to life domains, systems, and organizations that, as social structures, shape the context for the majority of one’s lifespan, thereby influencing individual health (4). In turn, humans, as shapers of their environment, influence the entire living environment through holistic thinking and actions (30). This highlights the reciprocal relationship between people and their environment.

To consider environmental domains and human-environment interactions comprehensively, we base our understanding of the environment on two models: The first is Barton and Grant’s (18) Health Map as introduced above, which represents the most significant framework for understanding health determinants in the field of public health. The Health Map has been extensively utilized in public health research (31). It effectively captures the dynamic interplay between individuals, communities, and their surrounding environments, recognizing a broad spectrum of determinants crucial to public health (31). However, Malsch (30) asserts that there is a paucity of consideration of individual social behavior arising from interactions with the physical environment, along with subjective environmental perception. Consequently, people themselves are regarded as a form of environment. Malsch (30) and Malsch et al. (32) therefore put forth a holistic approach that broadens the scope of environmental understanding to encompass the psycho-social environment and, consequently, the person-environment interaction as delineated in the Person-Environment Relationship Model (PERM) by Rauthmann (33). This approach is assumed to facilitate the coverage of all relevant upper categories of health determinants. According to Rauthmann (33), the *Psycho-Social Environment* is the product of the interplay between a person (e.g., cognition, behavior, characteristics, dispositions, affectivity, or narratives) and environmental variables (e.g., ecological phenomena, life events, situations, institutions, or culture), which compete, adapt, and influence each other over time and through interaction. The resulting outcome variable of the *Psycho-Social Environment* includes an individual’s personality, behavior, experiences, and perceptions. This comprehensive perspective, which encompasses both Barton & Grant (18) environmental dimensions and the psycho-social dimension, serves as a crucial foundation for the multidisciplinary approach of the SEHM. Consequently, it is utilized as a framework and rationale for our research.

Antonovsky (14), in contrast, defines behavior and experience as factors that trigger a *State of Tension* that the individual must cope with or that have a direct positive influence on the *Ease/Dis-ease Continuum* via the *SoC*. At this juncture, the link between human-environment interactions and their impact on health is immediately apparent. Nevertheless, the Health Map by Barton and Grant (18) lacks explicit consideration of these human-environment interactions defining the *Psycho-Social Environment* (see Table 1), even though psycho-social components are recognized by Antonovsky (14) as both resources and stressors.

**Figure 2:**
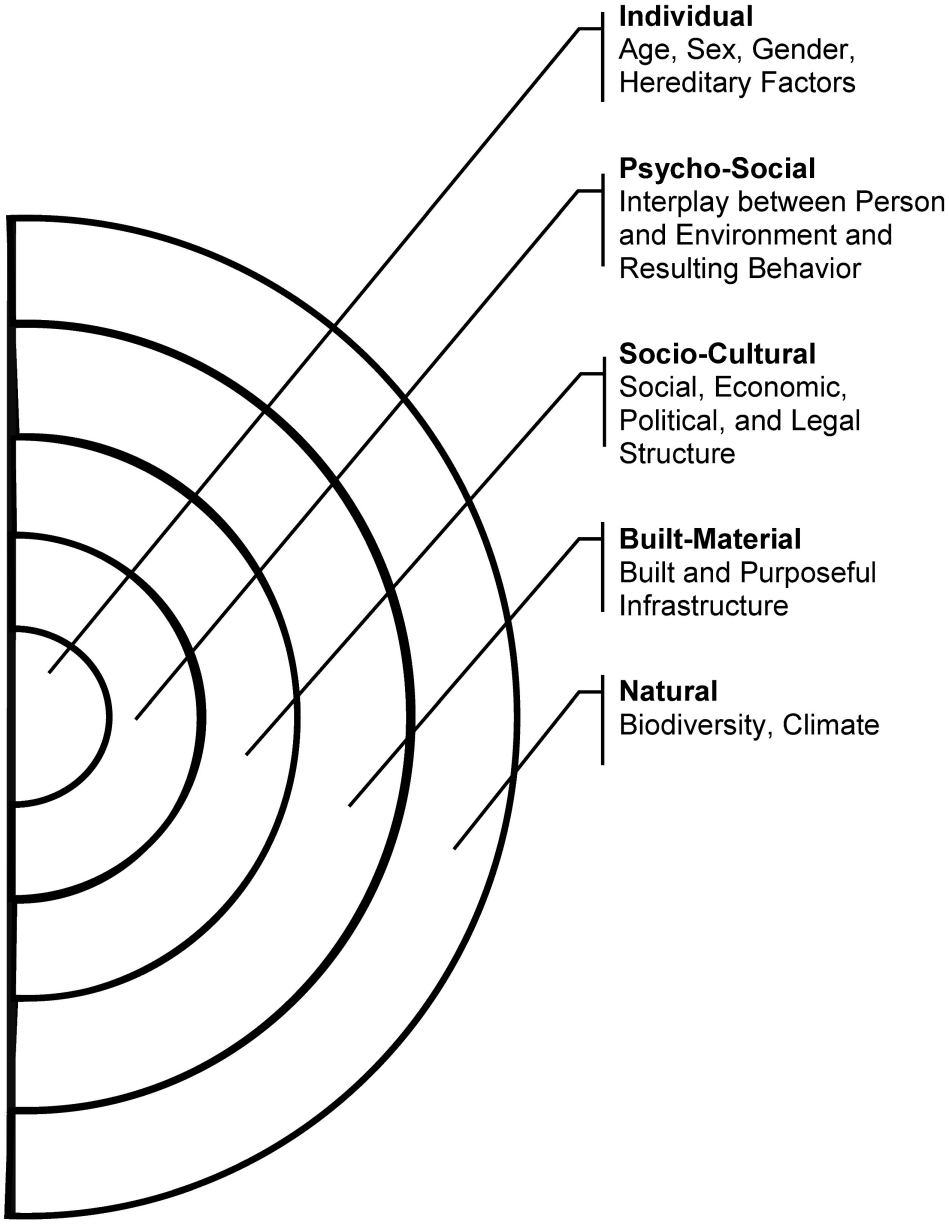
Environmental concept of the inquiry, including the dimensions of the human-environment interaction spectrum [Source: own illustration based on Barton and Grant (18), p. 252; Malsch (30) para. 24; Rauthmann (33), p. 437 ff.].

This lack of consideration highlights the need for the inclusion of the individual’s Psycho-Social Domain as a fifth environmental dimension in Barton and Grant’s (18) framework, which forms the analytical structure for further development of the Salutogenic Model. Moreover, considering human-environment interactions is essential for a comprehensive understanding of the genesis of health within a Salutogenic Model. Figure 2 presents our conception of the environment as an analytical framework that characterizes human-environment interactions and illustrates our understanding of the environment. The figure illustrates the four environmental dimensions defined by Barton and Grant (18), namely *Individual*, *Social-Cultural*, *Built-Material*, and *Natural* as well as their extension regarding the *Psycho-Social Dimension* based on Malsch (30), Malsch et al. (32) and Rauthmann (33).

### 1.3 Research objectives

An exhaustive analysis of where and how health is generated can only be achieved by placing human-environment interaction in focus, requiring a widened perspective on salutogenic explanatory models, which to date constitutes a research gap. Hence, the first objective of this paper is to augment Antonovsky’s Salutogenic Model (1979) based on human-environment interactions and the intricate environments hosting their determinants. To accomplish this, we use our conception of the environment as shown in Figure 2. As all dimensions interact and impact each other in determining the subject’s state of health, this research is devoted to delineating how Antonovsky’s Salutogenic Model can be expanded to integrate an understanding of human-environment interaction, thus addressing the identified issue of insufficient differentiation among environmental dimensions.

As the explanation of health genesis has evolved across various disciplines since the development of Antonovsky’s model in the 1970s, embedding the model extension into the current state of research is essential. Additionally, recognizing environmental dimensions as health determinants for a comprehensive understanding of health genesis presents an interdisciplinary challenge. As a result, explanations of health genesis occur within numerous models and theories, each describing health determinants from various disciplines. Nevertheless, no model to date integrates these different theories and models in the form of an interdisciplinary health explanation model. Closing this gap constitutes the second research objective. Based on a sampling approach (34), the objective of this study is to examine the potential for interdisciplinary models and theories to enhance Antonovsky’s (14) Salutogenic Model in the context of contemporary research. Rather than undertaking a comprehensive systematic review, the aim is to gain a deeper understanding through a critical narrative approach (35), thereby adopting a more holistic perspective on the genesis of health.

Objectives one and two will, in their synthesis, contribute to objective three: the development of an integrative *Salutogenic Environmental Health Model* (*SEHM*) as an extension of the Salutogenic Model, incorporating the diverse environmental dimensions and leveraging interdisciplinary explanatory approaches to explain the genesis of health. This endeavor constitutes the central research contribution of the paper. Positioned as an evidence-based operational level for health promotion, the SEHM aspires to comprehend the intricate pathways and influence levels involved in health explanations. In distinction from singular explanatory models concentrating on particular realms of health research, the SEHM is intended to offer a broader comprehension of the genesis of environmental health. While it refrains from asserting an exhaustive amalgamation of all existing models, the SEHM operates as an interwoven system acknowledging the genesis of health through human-environment interaction within the interdisciplinary research landscape.

## 2 Methodology

A methodological triangulation was used for the realization of the SEHM. Firstly, a Basic Model was developed, establishing a connection between the Salutogenic Model and environmental dimensions, and serving as the cornerstone for the model fusion. Subsequently, through a narrative literature review, fundamental interdisciplinary example models explicating health genesis from diverse perspectives were identified. Finally, these models were used in a theoretical analysis and synthesis process to identify gaps in the explanation of health development by the Basic Model. This was done to further develop the Basic Model by synthesizing all models into the SEHM.

### 2.1 Basic Model foundation

**Figure 3:**
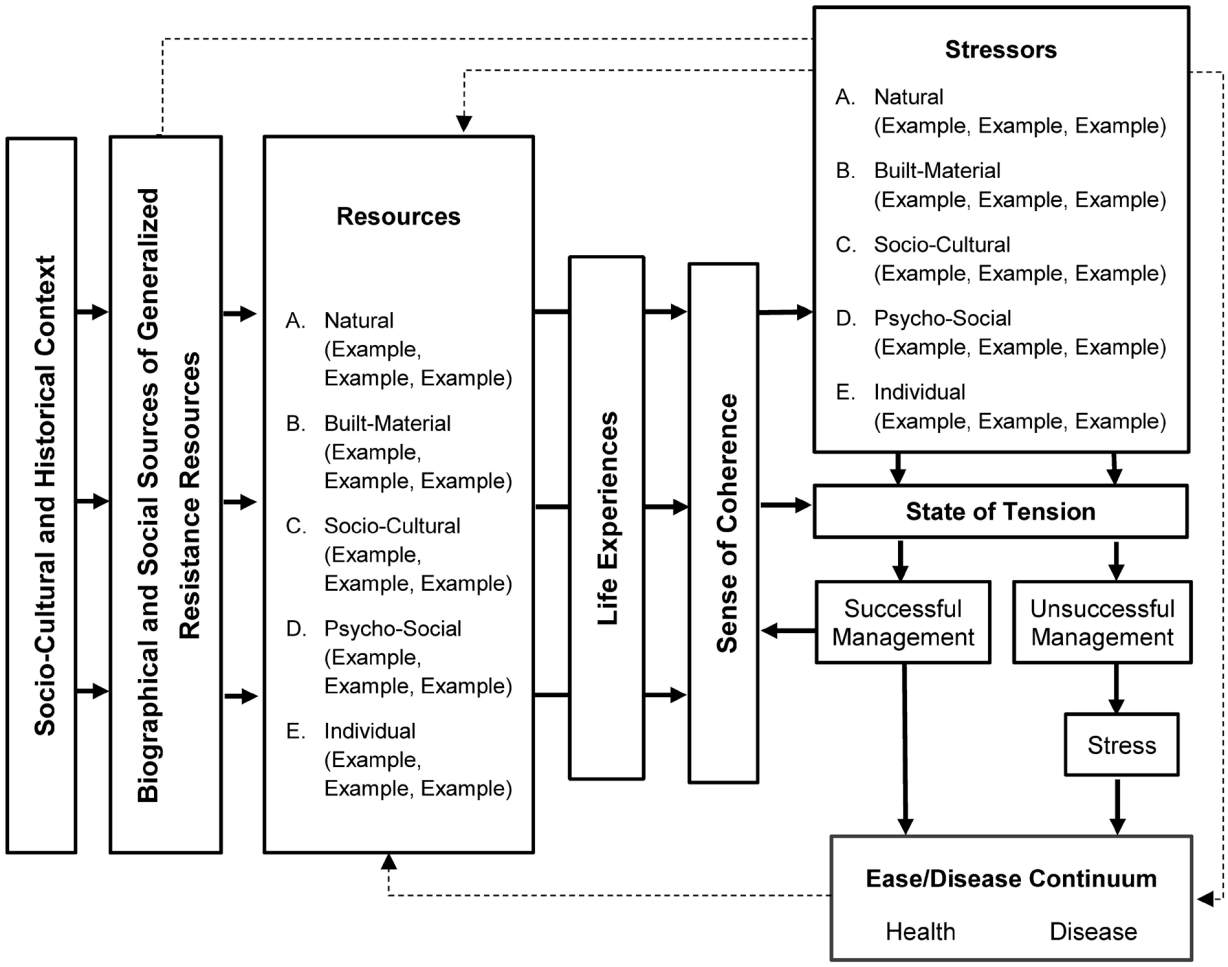
Basic Model (own illustration).

The Basic Model (Figure 3) was developed based on Antonovsky’s Salutogenic Model. The five environmental dimensions were integrated into the model to capture the concept of human-environment interaction (first research objective). Antonovsky’s *Stressors* and *GRRs* determinants were replaced by the A. *Natural*, B. *Built-Material*, C. *Socio-Cultural*, D. *Psycho-Social* and E. *Individual Dimensions* of the environmental conception described above to capture the complex determinants in a structured way.

### 2.2 Model identification—narrative literature review

After creating the Basic Model, it was expanded by further models from various disciplines to explain the development of health across disciplines more holistically. The goal of this research is not to provide a comprehensive review of all existing models. Instead, it aims to identify non-exhaustive samples of the interdisciplinary literature through the use of a sampling approach (34). By doing so, the goal is to clarify the diverse approaches through a critical lens, thereby establishing a conceptual foundation (36) for the development of a new salutogenic environmental health model.

In order to achieve this objective, a narrative, non-exhaustive literature search was conducted in July 2022 using the databases PubMed, Livivo, Psyndex, and Web of Science. The search was expanded through a snowballing search. To exemplify the notion of interdisciplinary models, a selection of well-known and pertinent models was made that pursue a collaborative approach through the integration of diverse perspectives, theories, and methodologies from various disciplines, thereby contributing to a comprehensive research methodology (37) (see Table 2). These include models from health psychology, environmental medicine, biology, and urban architecture, among others. The selection was made with great care, taking into account the research objectives and the relevance of the work for future developments in health promotion (36). This will contribute to a deeper understanding of health development with interdisciplinary perspectives (35). The authors are aware of the justified criticism of the selection bias of narrative reviews and address this in the discussion.

**Table 2:** Included models and theories.

Numeric code	Models and theories	Original research discipline
(1)	Salutogenic Model (14)	Health Science
(2)	Systemic Requirements-Resources (SAR) Model (47)	Psychology
(3)	PAKARA-Model (53)	Architectural Psychology, Urban Development
(4)	Ecosocial Theory (48)	Social Epidemiology
(5)	Attention-Restauration Theory (ART) (52)	Environmental Psychology
(6)	Social Determinants of Health (42)	Sociology, Social Epidemiology
(7)	The Ecological Circle (54)	Ecological Psychotherapy
(8)	Transactional Model of Stress (58)	Psychology
(9)	The National Institute on Minority Health and Health Disparities (NIMHD) Research Framework (43)	Health Equity Research, Social Ecology, Aging Research
(10)	Eight Classes of Key Factors (determinants’) that influence health status and quality of life (45)	Integrative Approach
(11)	Four Pillars of Health (44)	Integrative Approach, Health Science, Biomedical Science
(12)	Determinants of Health (84)	Disease Prevention, Health Promotion
(13)	Vulnerability-Stress-Model (61–63)	Clinical Psychology
(14)	Integrative Model of Salutogenesis (16)	Psychology; Health, Nutrition and Sports Sciences
(15)	Three-Dimensional Personality Model (46)	Psychology

To ensure the appropriateness of the selected models for the research objectives, the inclusion and exclusion criteria, as detailed in Table 3, were applied in a manner consistent with the research objective. They should be related to health, explain its development, and be based on a human-environment interaction perspective. For example, the Life Model (38) was excluded from consideration as it is aimed at health promotion interventions. Similarly, the concept of the Place of Identity (39) and its extension (40, 41) were excluded due to the absence of a direct reference to health by the authors.

**Table 3:** Eligibility criteria for interdisciplinary models.

Inclusion		Exclusion	
Key	Explanation	Key	Explanation
Models and Theories	Models and theories that imply an overall concept will be included.	Empirical Studies, Theoretical Thoughts	Literature that merely describes an approach or empirical findings on specific aspects of models or theories of health development without implying a concept that describes the genesis of health will be excluded.
Reference to Health	The models and theories focus on physical and/or mental health. At the very least, the authors must describe a reference to health.	Missing Reference to Health	If the authors do not reference health in terms of health implications, the model or theory will be excluded, even if the connection to health is apparent.
Explanation Models	Models and theories that explain the development of physical and/or mental health.	Intervention Models	Models and theories that are used exclusively to implement health promotion interventions.
Human-Environment Interaction	Models and theories should be based on a basic understanding of human-environment interaction (as a minimum, the basic assumption of the model/theory must understand the individual and, accordingly, health development as interacting with the environment).	Health-Related Models without Environmental Reference	Models and theories that do not establish an environmental reference in their description will be excluded.

In preparation for the descriptive and interpretative analysis (34), all models and theories included in the conceptualization (Table 2) were first broken down into single components and assigned to a category system with their definitions (taken from the original literature where possible; please see Supplementary Data Sheet 1). The category system was developed deductively in accordance with the theoretical framework described above. The defined understanding of the environment was employed, which encompasses the four environmental dimensions of Barton & Grant (18) and the extension proposed by Malsch (30). The category system (see Supplementary Data Sheet 1) thus comprised the five environmental dimensions (see Figure 2)—*Natural*, *Built-Material*, *Socio-Cultural, Psycho-Social*, and *Individual* - which were defined by the relevant literature. All components of the Salutogenic Model and all other considered models and theories were assigned to these five categories to systematically structure them and compare their fit with the assumed human-environment interaction. The full process can be inferred from the Supplementary material. For traceability to the original models and their components, these have been listed and numbered in the Supplementary Table 2.

### 2.3 Theoretical analysis and synthesis process

In the theoretical analysis process, the components of the various models listed in the Supplementary Table 2 were compared with those of the Basic Model. This was done to identify components that were not included in the Basic Model, thus revealing its gaps. The identified components were ultimately integrated into the Basic Model so that the gaps were closed. Through an iterative and recursive process of gap identification and closure, the SEHM was refined, as is usual for narrative reviews (34), and finally summarized and completed by synthesis. The subsequent analysis process is structured according to the gaps identified and the resulting expansion of the Basic Model, aggregated in the synthesis stage. This synthesis contains both descriptive and interpretative analyses (34).

#### 2.3.1 Lack of Context Conditions and the Resource-Stress-Continuum

The Social Determinants of Health Model by Schulz and Northridge (42), the National Institute on Minority Health and Health Disparities (NIMHD) Research Framework (43), the Four Pillars of Health by Patwardhan et al. (44), and the Eight Classes of Key Factors by Lawrence (45) are all models that categorize determinants of health from different contexts, which can be assigned to the environmental dimensions for this paper.

Drawing on sociology and social epidemiology, Schulz and Northridge (42) distinguish four interacting levels of determinants, including *Individual* and *Interpersonal Aspects* as well as *Social Contexts*. In addition, the *Natural* and *Built-Material Environments* are defined as levels of influence. The NIMHD framework by Alvidrez et al. (43) is an interdisciplinary matrix considering health-relevant levels (x-axis) and health-influencing domains (y-axis). It focuses on *Individual, Interpersonal*, and *Social-Societal Factors* and emphasizes the *Health System* as a separate influencing domain. Both models summarize many variables within their categories or components, which could be identified as *Stressors* and *Resources* and assigned to the environmental dimensions of the Basic Model. In addition, the models included further variables that were not considered to be either beneficial or detrimental to health. As a result, a value-free component (**Context Conditions**) needed to be included to address the challenge of appropriately categorizing factors initially understood as neutral circumstances.

The importance of such a component became apparent through consideration of the integrative approach of the Four Pillars of Health by Patwardhan et al. (44) and the Eight Classes of Key Factors by Lawrence (45). Both define core categories that can be allocated to the five dimensions of the environment and assign health variables to them in a value-free manner. Patwardhan et al. (44) highlight only the *Health System* as a support resource that comes into action when one of the four main pillars—*Nutrition, Lifestyle, Genetics*, and *Environment—*is weakened. Lawrence (45) argues that human health is influenced not only by the *Material* and *Non-Physical Variables* of the environment but also by the *Interrelationships* between them. Therefore, a separation of environmental variables and their context conditions is infeasible, whereby the basic understanding of human-environment interactions is underlined once again.

Based on these four models, a new component of **Context Conditions** was defined and divided into the five environmental dimensions. These environments are initially neutral contexts that contain value-free determinants, such as genetics, lifestyle, structural aspects, infrastructure, and climate. Determinants only become factors that positively or negatively influence health when characterized individually and transferred to *Resources* or *Stressors*. For instance, genetic conditions can give rise to genetic-constitutional resistance resources (*Resources*) or hereditary diseases (*Stressors*). The structural context can have a health-promoting effect with a functioning healthcare system or a health-damaging effect with inadequate healthcare. Similarly, nature can have a restorative impact on parks and bodies of water or cause disease through environmental hazards.

In light of this recognition of the continuum potential (see p. 4) of the environmental determinants, a multidimensional **Resource-Stress-Continuum** consisting of the five environmental dimensions was defined as a core component. Core components are here understood as theoretical concepts that can exist independently, but form a conceptual model when considered together. The aforementioned core component of the **Resource-Stress-Continuum** comprises the initially value-free determinants of the *Natural, Built-Material, Socio-Cultural, Psycho-Social,* and *Individual Dimension*. The individual health-promoting or health-damaging properties of these determinants along the continuum have the potential to lead to *Positive Life Experiences* or trigger a *State of Tension* in Antonovsky’s sense, ultimately influencing human health.

#### 2.3.2 Lack of differentiation between the Psycho-Social Dimension and the Active Individual

Personality traits and behavior were assigned to the *Psycho-Social Environment*. According to the PERM (33), which was used to define the *Psycho-Social Environment* in the conception of the environment, the psycho-social outcome variable results from the interaction between the person and the environmental dimension. It concerns the personality and implies a person’s behavior (lifestyle, reactions) and experience or perception (33). Antonovsky, and thus the Basic Model, lists the response to *Stressors* as a separate component or concept (*Individual Processing*). The Three-Dimensional Personality Model (46) describes the unconscious processes involved in personality development, and the Integrative Salutogenic Model (16) points out the individual as an active being. Accordingly, it was necessary to differentiate the *Psycho-Social Environment* to meet the complex requirements of the different models.

Gebhard (46) describes the development of individual personality structure and its influence on mental health in the context of environmental psychology and with reference to the Three-Dimensional Personality Model. The model assumes that human personality develops through self-experience and engagement with “things,” i.e., through the structure of interaction between humans and the non-human environment (46). Accordingly, the characteristics of a subject originate not only from their type but also from their environment. The model’s understanding aligns precisely with the definition of the *Psycho-Social Dimension* and is therefore assigned to it. Like other environmental dimensions, which contain variables as passive conditions, the developed personality is also a passive or unconscious determinant that indirectly influences health through *Individual Processing*.

In contrast to the passive individual, the *Active Individual* in the Integrative Salutogenesis Model can consciously influence their health through health-related activities. Health-related activities arise from an interplay of *External* (social-interpersonal, socio-cultural, material) and *Internal* (personal-psychological, physical) *Resources* (16). This highlights the interplay of *Environmental Conditions* and the *Individual Level*, which implies the human-environment interaction. By adopting specific health behaviors, such as maintaining a healthy diet, regular exercise, refraining from smoking and heavy alcohol consumption, and taking advantage of preventive examinations as well as early detection measures, individuals can actively influence their state of health (16). To emphasize its direct influence on health, it appeared reasonable to include behavior separately in the SEHM.

Faltermaier et al. (16) argue that health behavior should be integrated into an individual’s daily routine of people and that it is associated with their personal world and way of life. *Behavior* and *Lifestyle* were subsumed under the **Active Individual** and integrated into the *Psycho-Social Dimension* of the SEHM as a separate sub component.

In the Integrative Salutogenic Model (16), health perceptions, awareness and cognitions, and personal identity are not actively controlled by the individual. These determinants provide subjective references and are linked to individual goals (16). They relate to the individual’s self and are in relation to the environment. Thus, they belong to the general passive *Psycho-Social Dimension*. The Integrative Salutogenic Model demonstrates that both the internal psychological processes or cognitions and the externalized active behavior are influenced by the interaction between a person and their environment and can have a direct or indirect impact on their health. Finally, the SEHM distinguishes the *Psycho-Social Dimension* into passive and active determinants. The active determinants, such as *Behavior* and *Lifestyle*, are characterized as the ‘**Active Individual’** sub component, directly impacting the *Health Continuum*. Faltermaier et al. (16) defined the *Health Continuum* as a range between maximum and minimum health. He expands the salutogenic perspective of Antonovsky’s model by eliminating the concept of illness based on his criticism of the lack of a salutogenic perspective. This extension was also adopted for the SEHM, thus establishing the *Ease/Disease Continuum* as the **Health Continuum**.

#### 2.3.3 Lack of Mutual Interaction within the Resource-Stress-Continuum and the transition to Processing

The newly defined *Resource-Stress-Continuum* (see section 2.3.1) prompts inquiry into the interaction between its sub components (*Resources, Context Conditions, Stressors*). According to Antonovsky (14), *Stressors* create *Tension* and *Resources* can contribute to *Successful Tension Management* based on a strong *Sense of Coherence*. However, the interaction of the sub components remains unclear and is further differentiated using the Systemic Requirements-Resources (in German: Systemisches Anforderungs-Ressourcen-Modell; SAR-) Model (47) as well as the Eco-Social Theory (48, 49). Antonovsky’s *State of Tension* is characterized as a disturbed balance of the organism triggered by *Internal* or *External Demands* (14). According to the SAR-Model, *Demands* arise from the environment’s and human beings’ mutually influencing systems (i.e., for the SEHM from the interaction of the dimensions). *Demands* are met with the help of *Internal* (Individual Dimension) and *External* (Environmental Dimension) *Resources* (50) and influence the state of health depending on the degree of success in coping with them (47, 51). Accordingly, health depends on the interaction of *Resources* and *Stressors*.

Krieger’s Eco-Social Theory (1994, 2019) also includes the *Cumulative Interplay* of susceptibilities, resistances, and disease exposure. The interplay of the three sub components results from life-long experience and influences health (49). For the Basic Model, this results in a further component encompassing the **Mutual Interaction** of all sub components of the *Resource-Stress-Continuum*.

The SAR-Model and Eco-Social Theory describe the effect of the interplay on the *Health Continuum* by further mediating variables of the *Processing* procedure. For the Basic Model, this leads to the problem of the pathways of action between Antonovsky’s *State of Tension*, the **Mutual Interaction**, and the *Processing* procedure, which is addressed by the Attention-Restoration Theory (52)—hereafter called ART—, the PAKARA-Model (53), and Willi’s (54) Ecological Psychotherapy.

The models show that the *State of Tension* is not simply triggered by *Stressors*, but must be understood as a lack of balance between stressors and resources. The ART suggests that a lack of balance can lead to detrimental health risks. This means that overuse of *focused attention*—necessary for processing mentally demanding tasks, focusing and avoiding distractions, and regulating inappropriate feelings and actions—leads to *Fatigue* and *Stress* (55). For example, *Attention* can be overtaxed by urban environments and lifestyles (52) and restored by sleep, meditation, or *Restorative Environments* (parks, lakeside). As a result, *Fatigue* and *Stress* can be reduced (21, 52). In other words, the *Imbalance* between the *Requirements* on *Attention* (stressors) and the *Potential to Restore* the ability to concentrate (resources) is a *State of Tension* that must be managed by the individual.

The PAKARA-Model (53) of architectural psychology and urban development also incorporates basic human needs to understand individual *Imbalance*. Their satisfaction is individual and influenced by external urban architectural factors, divided into health-promoting (resources) and health-damaging (stressors) directions of action. The basic needs for *Privacy*, *Identification* and *Stimulation* can take on a pathological value due to *Oversaturation* or *Undersaturation* (53). For example, healthy stimulation can lead to hyperstimulation through oversaturation, resulting in sensory and neurological overload, which is associated with stress and mental illness (56). In contrast, a *Balance* between the two poles of *Oversaturation* and *Undersaturation* has a health-promoting effect (53). It follows that **Imbalance** and **Balance** of *Stressors* and *Resources* resulting from **Mutual Interaction** are included as further core components within the SEHM.

**Excursus**: Willi (54), in his approach to Ecological Psychotherapy, also emphasizes the *Basic Areas of Personality* as needs to be satisfied. A considerable health risk is therefore associated with a persistent lack of negative or distorted response to the *four personality areas*—ego function, reality testing, self-esteem and identity—which are created by shaping one's own environment and generating effects (54). The development of the responding action takes place within the framework conditions, i.e. the environment and the surroundings. Surroundings and environment are initially passive conditions that surround the individual. It is only through the shaping of one's own environment and the generation of effects that the basic areas of personality are created. Basic needs therefore arise from the interaction between person and environment and are embedded into the *Psycho-Social Dimension*, but are essential for **Balance** and **Imbalance**.

The PAKARA-Model is also based on the assumption that environmental influences do not affect health directly, but indirectly via unconscious perception and evaluation (53). Accordingly, psychological theories such as the Vulnerability-Stress-Model and the Transactional-Stress-Model—described in the following chapter—and thus also the Salutogenic Model, are used to explain the processing of the unbalanced poles of influence on *Oversaturation* and *Undersaturation*. According to the Salutogenic Model, the **Balance** between under and overloading, the *Successful Management* of the *State of Tension* (**Imbalance**), leads to *Positive Life Experiences* (14), which results in a *Sense of Coherence* as a central health-promoting component (57). In summary, this means that *Resources, Context Conditions;* and *Stressors* as the sub components of the *Resource-Stress-Continuum* are understood to interact with each other to satisfy the basic human needs contained therein and arising from them. *Positive Life Experiences* and the corresponding health-promoting *SoC* result from the equilibrium of a balanced interaction. An unbalanced interaction can be understood as an **Imbalance**, i.e., a state of tension that needs to be processed by the individual.

#### 2.3.4 Lack of processing connections and Physical Processing

According to Antonovsky’s Salutogenic Model, the interaction between environmental factors and *Stress* can create an *Imbalance* that replaces the *State of Tension*. The way individuals process *Stressors* determines whether environmental factors are beneficial or detrimental to health (14). The author utilized stress theories, such as Lazarus and Folkman’s (58) Transactional-Stress-Theory, to explain the *Processing* procedure. The SEHM incorporates and analyses stress theories in detail to identify pathways between the core components of *Mutual Interaction*, *Imbalance*, and *Physical Processing*, which are newly acquired in the SEHM.

The Transactional-Stress-Model, developed by Lazarus and Folkman (58), describes the *Psychological Process* between an incoming *Stimulus* (stressor) and the potential stress reaction. Whether *Stress* with its harmful potential ultimately arises, depends on the individual’s *Cognitive Evaluation* and *Coping* mechanisms (59). In other words, *Stress* is a result of *Individual Processing*. *Cognitive Evaluation* is divided into *Primary* and *Secondary Assessment*, the combination of which determines whether *Stress* arises. The *Primary Evaluation* assesses the significance and consequence of the *Stimulus*, while the *Secondary Evaluation* assesses the available *Coping* resources (59, 60). The SEHM features a **declining arrow** from *Mental Processing* to *Imbalance*, which illustrates the *Primary Assessment* and a **two-sided arrow** for *Mutual Interaction* to emphasize the *Secondary Assessment*. The assessment is followed by *Coping*, which involves handling the discrepancy between perceived demands and resources (59). When transferred to the SEHM, it can be understood as assessing the *Imbalance* resulting from the *Mutual Interaction*. The Vulnerability-Stress-Model, as described in Roch and Hampel (61), Wittchen and Hoyer (62) or Wirtz (63) from clinical psychology, among others, highlights the importance of *Coping, Resilience, Vulnerability*, and *Stress* in developing mental illnesses. The model proposes that the onset of mental illness is contingent upon the *Interplay* of these four components. The model addresses the *Interplay* between *Resources* and *Stressors* and emphasizes the importance of *Coping* and *Resilience*, which are interrelated. Accordingly, an arrow must be added from the *Mutual Interaction* to *Coping* or *Mental Processing* and the *Sense of Coherence*.

The inclusion of Krieger’s (48), Eco-Social Theory revealed a further gap in the *Processing* procedure. The social epidemiologist developed the central concept of *Embodiment* on the basis of her research into the links between group-specific diseases and social inequalities. Krieger (49) utilizes the concept of *Embodiment* to describe the physiological incorporation of the *Social* and *Material Environment* resulting from a social and ecological context and its impact on population rates and health distribution (64, 65). The **Physical Processing** of the *Imbalance* was correspondingly added to the Basic Model and is correspondingly featured in the SEHM.

#### 2.3.5 Synthesis

Based on the analysis of the interdisciplinary models, gaps and resulting components describing health genesis were identified and summarized in Table 4.

**Table 4:** Identified gaps and core components in the Basic Model.

Core components of the Basic Model	Further core components for the SEHM	Numeric code
Socio-Cultural and Historical Context	Resource-Stress-Continuum	I.
Biographical and Social Sources of Generalized Resistance Resources		
Resources (Natural, Built-Material, Socio-Cultural, Psycho-Social, Individual)		
Stressors (Natural, Built-Material, Socio-Cultural, Psycho-Social, Individual)		
	Mutual Interaction	II.
	Balance	III.
Life Experience	Positive Life Experiences	IV.
Sense of Coherence	Sense of Coherence	V.
State of Tension	Imbalance	VI.
	Physical Processing	VII.
	Mental Processing	VIII.
Successful Management	Successful Management	IX.
Unsuccessful Management	Unsuccessful Management	X.
Stress	Stress	XI.
Ease/Disease Continuum	Health Continuum	XII.

Six new components for a SEHM were derived, which were either created by modifying or extending components of the Basic Model (**Resource-Stress-Continuum, Imbalance, Mental Processing**) or were not present in the Basic Model (**Mutual Interaction, Balance, Physical Processing**). In conjunction with the original components of the Basic Model, this process yielded a total of twelve core components for the SEHM.

In this context, core components (Table 4) refer to theoretical concepts that can stand alone, but together form a conceptual model. These concepts can be further differentiated and explained by sub components (Supplementary Table 1). For instance, the analysis process identified a *Resource-Stress-Continuum* as a core component, comprising three sub components: *Resources*, *Context Conditions*, and *Stressors*. Each sub component distinguishes five environmental dimensions (*Natural, Built-Material, Socio-Cultural, Psycho-Social, Individual*) that contain variables affecting health. Variables are understood to be the smallest units of factors that influence health, such as age, diet, social class or the environment in which people live.

**Figure 4:**
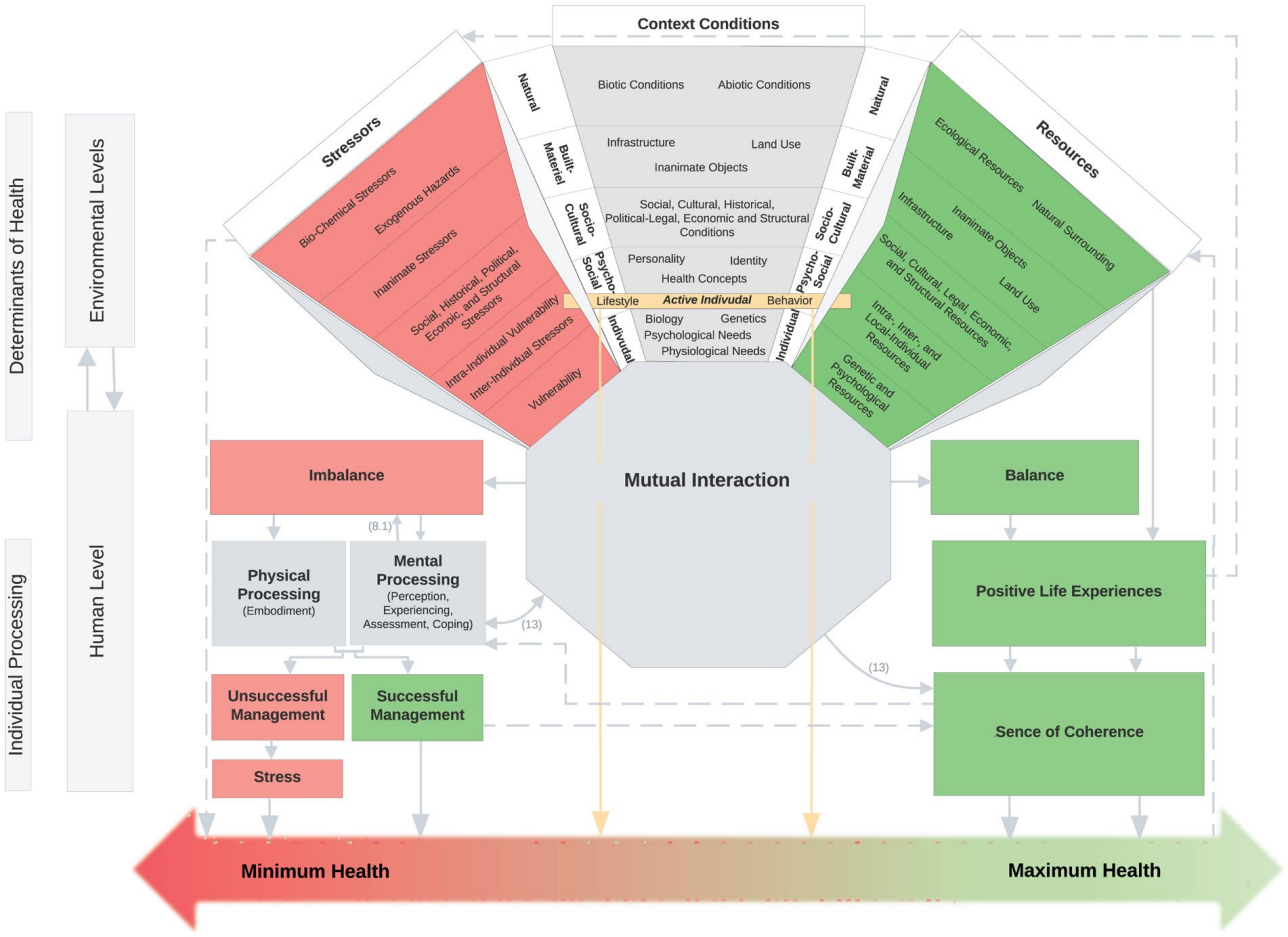
Salutogenic Environmental Health Model. 12 core components (Resource-Stress-Continuum, Mutual Interaction, Imbalance, Physical Processing, Mental Processing, Unsuccessful Management, Successful Management, Stress, Balance, Positive Life Experience, Sense of Coherence, Health Continuum); sub components within the Resource-Stress-Continuum (Stressors, Context Conditions, Resources); coloring: harmful components in red; health-promoting components in green; neutral components in gray; active individual in yellow.

The twelve core components, respectively, their sub components were used to assign all variables of the interdisciplinary models (see Supplementary Table 2). In order to gain a more comprehensive understanding, the various variables associated with the different components were subjected to an inductive categorization process. Variables with content that was thematically similar across different models were grouped together into key determinants, which can be observed in the SEHM visualization (Figure 4) and can be traced through their listing in the first table of the Supplementary material.

## 3 Proposed Salutogenic Environmental Health Model

The final Salutogenic Environmental Health Model is presented in Figure 4, with all core and sub components listed in the Supplementary Table 1 and explained by the components and variables of the fifteen integrated interdisciplinary models. Please direct your attention to the footnotes in Supplementary Table 2, which provide an explanation of the individual models.

Figure 4 presents the SEHM, which depicts the twelve core components situated at the environmental and human levels and their interaction. The core component of the multidimensional **Resource-Stress-Continuum,** which contains the three sub components **Resources**, **Context Conditions** and **Stressors**, is also shown within the environmental dimensions, which contain the various determinants of health. The mapped determinants were obtained by extracting the variables from the various interdisciplinary models and inductive categorization (described in the synthesis section). Their differentiation is shown in Supplementary Table 1. The **Active Individual**, and the influence of it on the **Health Continuum**, receives particular emphasis at the *Psycho-Social Level*. This level is the result of an interaction between the environment and the individual. At this level, the SEHM distinguishes between active and passive determinants. The active determinants are those that can be actively controlled by the individual, as behavior and lifestyle. In contrast to the active determinants of the **Active Individual,** the passive determinants of the environmental dimension exert an indirect effect on the **Health Continuum** via their **Mutual Interaction** and their **Individual Processing** at the human level. The *Mutual Interaction* of *Resources*, *Context Conditions* and *Stressors* can, provided it is in **Balance**, result in *Positive Life Experiences* and contribute to a strengthening of the **Sense of Coherence**, which consequently has a health-promoting effect. However, if the three sub components are not in balance, there is a state of tension (**Imbalance**) that can be explained by the oversaturation or undersaturation of basic needs. The *Imbalance* is ultimately processed through *Perception, Experience, Assessment* and *Coping* on a mental level (**Mental Processing**) or through the *Embodiment* on a physical level (**Physical Processing**). **Unsuccessful Management** of the processing process leads to **Stress**, which has a detrimental effect on health and therefore leads to the pole of minimal health. **Successful Management**, on the other hand, contributes to a stronger *Sense of Coherence* and has a health-promoting effect, so that the state of health moves toward maximum health on the continuum. The SEHM conceptualizes health as a continuum, adopting a salutogenic perspective that moves away from a pathogenesis-focused approach. This redefinition of health no longer situates it within the poles of health and illness, but rather between minimum and maximum health.

Supplementary Table 1 elucidates the twelve core components and the sub components within the *Resource-Stress-Continuum*. The color coding is based on the visualization of the SEHM, whereby health-promoting components are depicted in green, health-damaging components in red, and neutral components in gray. The *Active Individual* is highlighted in yellow. The sub components of the *Resource-Stress-Continuum* (*Context Conditions, Resources, Stressors*) each comprise the five environmental dimensions (*Natural, Built-Material, Socio-Cultural, Psycho-Social, Individual*) and their health determinants. These determinants were derived as part of the synthesis process (see above) from the inductive categorization, which was based on the quantity of individual variables of the interdisciplinary models. The original model of the individual variables contributing to the explanation of the health determinant, or the core and sub component, can be traced via the respective footnote in the Supplementary Table 2.

## 4 Discussion

The objective of this paper was to extend Antonovsky’s fundamental Model of Salutogenesis (14, 66), based on an understanding of human-environment interaction and taking into account interdisciplinary models as examples, to address the identified problem of insufficient differentiation between environmental dimensions and the determinants embedded in them, and to conceptualize the emergence of health in an interdisciplinary way, based on the current state of research.

The Basic Model, developed by incorporating the *Natural, Built-Material, Socio-Cultural, Psycho-Social, Individual Dimension* into Antonovsky’s Salutogenic Model, serves as the foundation of the analytical process. The SEHM combines fifteen exemplary models from various scientific disciplines to explain health development from a more holistic perspective. The formulation of the SEHM was informed by the identification of gaps in the Basic Model. These gaps were uncovered and closed through the exemplary inclusion of interdisciplinary models [sampling approach, see (34)] and the application of theoretical analyses with a critical approach (36). This approach facilitated an iterative conceptual development of the SEHM.

The original eleven components of the Salutogenic Model were expanded to twelve, with the addition of new components such as *Mutual Interaction, Balance*, and *Physical Processing*, and modifications to existing components such as the *Resource-Stress-Continuum*, *Imbalance*, and *Mental Processing*. The extension of Antonovsky’s Salutogenic Model to the SEHM resulted in a far more complex model. Models are typically intended to simplify complex theories or phenomena (67). However, this should be considered in the context that our world has become increasingly complex since Antonovsky’s model was developed, and that the state of knowledge on the genesis of health has also become more complex due to the intertwining of different explanatory approaches from multiple disciplines. In light of the vast array of variables that emerged during the breakdown of the models (see Supplementary Table 2), and the subsequent data reduction through category formation, a simplification of the current state of knowledge was achieved. This resulted in a model that contributes to a deeper understanding of the complex development of health and disease with an environmental public health perspective, with special consideration of human-environment interaction.

By integrating diverse explanatory approaches, the SEHM links the various processes involved in the development of mental and physical health, enabling a multifaceted understanding of these processes. It is crucial to acknowledge that this SEHM is not exhaustive, as it incorporates only a selected number of models, based on a non-exhaustive narrative literature review in accordance with the sampling approach (34). The authors are aware that the narrative nature of the review means that the selection of models is not reproducible and that there is a risk of selection bias. However, the focus here is on the conceptual contribution of individual literature and the necessarily subjective interpretation of individual elements (36). The interpretative methodology is necessarily perspectival, whereby the work here attempts to contribute to the quality of interpretative research through a transparent reflection of the limitations (35). Consequently, the resulting products are only “the starting point for further evaluation, not an endpoint in itself” (36) (p. 97). Therefore, subsequent testing of the SEHM is required in future research. A particular focus should be on investigating the causal relationships between the individual main components of the SEHM.

To further improve the validity of the model, a comprehensive systematic review should prospectively be conducted using interdisciplinary databases and sources to identify further interdisciplinary models. Future systematic research can also level out the tendency toward the psychological model inclusion. Other disciplines, such as medicine, social work, and biology, can be added until the content is saturated and the interdisciplinary fields targeting the genesis of health are represented. Acknowledging this limitation, future research should address to what extent the SEHM would have reached other conclusions if different models had been included.

The development of the SEHM presented challenges in certain areas due to varying definitions of component names and categorization within their respective models. This was particularly evident in the environmental dimensions of the *Resource-Stress-Continuum*. Given the interaction between humans and the environment, the five dimensions are interrelated and mutually influence each other, impeding an unambiguous categorization. Different categorization structures may also be possible, whereby it is to be tested how these would shape the model.

One significant advantage of the integrated SEHM is its fundamental integration of environmental dimensions into the Salutogenic Model. The complexity of health determinants within various environmental spheres and their relationships and impact pathways in the process of health genesis is recognized this way. However, the SEHM does not consider the digital environment as a distinguished category. This is because research still lacks models that connect the genesis of health to the digital environment. The WHO recognizes access to digital technology and the internet as a social determinant of health (68). Digital environments can have both positive and negative effects on health, for instance, by enhancing monitoring, diagnosis, and information sharing (69, 70). Improving access to public services, reducing bureaucracy through AI-supported technologies (71, 72), and enhancing the quality of life of oncology patients through digital health applications (73), can also significantly influence health results. At the same time, new digital environments such as the Metaverse pose questions on the future human-environment interaction, highlighting the need to investigate this dimension in more detail (74). For this reason, further research including the development of appropriate models is necessary to integrate this digital dimension into the SEHM in an evidence-based manner.

In addition to the digital aspects, animals and their effects on health, such as the reduction of loneliness through pet ownership (75, 76) or the development of zoonoses (77), were not taken into account due to the limited selection of models. This is particularly evident when considering approaches such as Planetary Health, EcoHealth, and One Health. These approaches are based on the assumption of the equivalence and interdependence of living human and non-human organisms, as well as the nature or ecosystems that surround them (78, 79). Based on principles such as systems thinking, sustainability (80), and health equity (81), these concepts serve as holistic perspectives and narratives that require interdisciplinary and participatory collaboration (82). These approaches encompass a large number of models. Therefore, they have not been included in the SEHM as a comprehensive approach. For future research, it is important to include models based on Planetary Health, Eco- or One Health approaches. For instance, the Planetary Health Framework proposed by Brousselle and McDavid (83), or the One Health Umbrella suggested by Lerner and Berg (82), could be considered.

Although the SEHM can explain health genesis with an environmental focus in an exemplary manner, it cannot provide explicit recommendations for promoting health. The Integrative Model of Salutogenesis by Faltermaier et al. (16) and the PAKARA-Model by Vollmer et al. (53) offer intervention theoretical approaches to health promotion beyond their explanatory theoretical components.

While the practical application of SEHM in health promotion requires the integration of the intervention theoretical aspects of these models and the consideration of interdisciplinary intervention models, SEHM can contribute toward an interdisciplinary understanding of health development and enable more holistic health promotion. In the context of health promotion interventions, the SEHM enables an overview of the five different environmental dimensions and the determinants contained within, which influence health. In addition, both the mental and the physical processing of these determinants and the components associated with them can be considered, including their interactions, which play a role in the development of health. Due to the comprehensive approach of the SEHM, health promotion can address all components of the model. This enables the consideration of the larger, holistic process of health development. Nevertheless, the SEHM serves as a launching pad for further research, which implies, in particular, the testing of the model.

## Data Availability

The original contributions presented in the study are included in the article/Supplementary material, further inquiries can be directed to the corresponding author.
