# Corrigendum: Salutogenic Environmental Health Model—proposing an integrative and interdisciplinary lens on the genesis of health

**DOI:** 10.3389/fpubh.2025.1546056

**Published:** 2025-01-28

**Authors:** Jule Anna Pleyer, Laura Dominique Pesliak, Annette Konstanze Fides Malsch, Timothy McCall

**Affiliations:** ^1^Department of Environment and Health, School of Public Health, Bielefeld University, Bielefeld, Germany; ^2^Joint Institute for Individualisation in a Changing Environment, University, Münster and Bielefeld, Germany; ^3^Department of Sustainable Environmental Health Sciences, Medical School OWL, Bielefeld University, Bielefeld, Germany

**Keywords:** alutogenesis, environment, health model, Antonovsky, interdisciplinary, theoretical analysis, determinants, health promotion

In the published article, there was an error in the author list, and author “Annette Konstanze Fides Malsch” was erroneously excluded. The corrected author list appears below.

Jule Anna Pleyer^1^, Laura Dominique Pesliak^1^, Annette Konstanze Fides Malsch^1,2*^^†^ and Timothy McCall^1,3*^^†^

In the published article, there was an omission of the affiliations for Annette Konstanze Fides Malsch. As well as having affiliation(s) [1], they should also have [*University of Münster and Bielefeld, Germany*].

The original article has been updated and the correct affiliation listing appears below:

^1^Department of Environment and Health, School of Public Health, Bielefeld University, Bielefeld, Germany

^2^Joint Institute for Individualisation in a Changing Environment, University, Münster and Bielefeld, Germany

^3^Department of Sustainable Environmental Health Sciences, Medical School OWL, Bielefeld University, Bielefeld, Germany

In the published article, there was an error in the Correspondence section. The corrected Correspondence list appears below.

^†^These authors contributed equally to this work and share last authorship

^*^ **Correspondence:**

Timothy McCall, timothy.mc_call@uni-bielefeld.de

Annette Konstanze Fides Malsch, a.malsch@salnas-institut.com

In the published article, there was an error in the **Funding** statement and some funding information was omitted.

Original Funding statement:

The author(s) declare that financial support was received for the research, authorship, and/or publication of this article. This research was funded within the program “Healthy Places—Therapeutic Landscapes” by the Peter Beate Heller-Stiftung of the German Stifterverband (project number: T0160/33738/2019/kln) and we acknowledge the support for the publication costs by the Open Access Publication Fund of Bielefeld University and the Deutsche Forschungsgemeinschaft (DFG).

The correct Funding statement appears below:

**FUNDING**

“The author(s) declare that financial support was received for the research, authorship, and/or publication of this article. This research was funded from 2021 to 2023 by the Chair in Environmental Health, Prof. Dr. Annette Konstanze Fides Malsch. The research was also funded from 2023 to 2024 by the visiting professorship Dr. Timothy Mc Call in the department of Environment and Health, at the School of Public Health, Bielefeld University and within the program “Healthy Places—Therapeutic Landscapes” by the Peter Beate Heller-Stiftung of the German Stifterverband (project number: T0160/33738/2019/kln) at the department Sustainable Environmental Health Sciences, Medical School OWL, Bielefeld University. We also acknowledge the support for the publication costs by the Open Access Publication Fund of Bielefeld University and the Deutsche Forschungsgemeinschaft (DFG).”

In the published article, there was an error in the **Acknowledgements** section. The corrected Acknowledgments appears below:

**Acknowledgments**

The authors are particularly grateful to Ann-Kathrin Lange for the preparatory work. The authors used the following AI tools: English grammar: Grammarly, English expression: ChatGPT & DeepL and the Research tool: Elicit.

In the published article, there was an error in the Author Contribution section. The corrected Author contributions statement appears below:

**Author contributions**

JP: Conceptualization, Data curation, Formal analysis, Investigation, Methodology, Resources, Visualization, Writing – original draft, Writing – review & editing. LP: Conceptualization, Resources, Visualization, Writing – original draft, Writing – review & editing. AM: Supervision, Conceptualization, Methodology, Visualization, Writing – review & editing, Project administration. TM: Funding acquisition, Writing – review & editing, Project administration.

The authors apologize for this error and state that this does not change the scientific conclusions of the article in any way. The original article has been updated.

