# Supplementary material for: Salutogenic Environmental Health Model—proposing an integrative and interdisciplinary lens on the genesis of health
Source: Front Public Health. 2024 Oct 17;12:1445181. doi: 10.3389/fpubh.2024.1445181 (PMC11524910; doi:10.3389/fpubh.2024.1445181)
Supplement: Supplementary file 1 [file Table_1.docx]

Supplementary Material

Table 1. Core and Sub Components *of the Salutogenic Environmental Health Model Including Explanation Using the Variables from Integrated Interdisciplinary Models and their Footnote Reference to Table 2.
12 core components (Resource-Stress-Continuum, Mutual Interaction, Imbalance, Physical Processing, Mental Processing, Unsuccessful Management, Successful Management, Stress, Balance, Positive Life Experience, Sense of Coherence, Health Continuum); Sub Components within the Resource-Stress-Continuum* *(Stressors, Context conditions, Resources); Five Environmental Dimensions within the Sub Components and the Health Determinants embedded in them; Coloring: Harmful components in red; Health-promoting components in green; Neutral components in grey; Active Individual in yellow.*

| **Numeric Code** | **Core and Sub-Components** | **Explanation** |
| --- | --- | --- |
| **I** | **Resource-Stress-Continuum** | Consists of the following: Contextual Conditions, Resources, and Stressors |
|  | **Context Conditions** | |
|  | **A. Natural Environment** | |
|  | Abiotic Conditions^4.2^ | Climate^6.1, 12.5^, Topography^6.1^ |
|  | Biotic Conditions^4.2, 7.6^ | Plants^12.5^ |
|  | **B. Built-Material Environment** | |
|  | Inanimate Objects^7.6^ | Buildings^6.2, 12.5^ (Housing^6.2, 9.5, 12.5^, Schools^6.2, 12.5^, workplaces^6.2^), Home Environment^9.5,12.5^, Community Environment^9.5, 12.5^, Physical Barrieres^12.5^, Construction Sites ^12.5^, Aesthetic Elements (Good Light, Benches)^12.5^ |
|  | Infrastructure | Transportation Systems^6.2, 12.5^, Services^6.2^ (Shopping, Banking, Health Care Facilities)^6.2^, Waste Transfer Stations ^6.2^, Sanitation^11.4^ |
|  | Land Use | Industry^6.2^, Recreational Environments ^12.5^ |
|  | **C. Socio-Cultural Environment**^14.2^ | |
|  | Social Conditions | Social Class^7.5^, Social Sources of Generalized Resistance Resources^1.11, 14.1^ (Social justice^6.1^, Social Networks^9.3^, Social Institutions^6.1^), Social Deprivation^10.6^, Investment and Capacity of the Community (Economic Development, Maintenance, Police Services)^6.2^, Family/Peer Norms^9.3^ |
|  | Cultural Conditions | Culture^7.5, 9.3^, Home Country^7.5^, Language^7.5^, Societal Values^6.1, 7.5^, Norms^9.3, 12.5^, Attitudes^6.1, 12.5^, Rules of Behavior^7.5^, Community Norms^9.3^, Cultural Institutions^6.1^ |
|  | Historical Conditions | Historical Conditions^1.10, 6.1^ |
|  | Political-Legal Conditions | Political Circumstances ^6.1, 7.5^ (Democracy^6.1^, Policymaking^6.2, 12.4^, Laws^9.2^, Legal Codes^6.1^), Policies^9.2, 10.5^ (Public, Fiscal, Ecology, Workplace ^6.2^, Health Care^9.4^), Political Influence^6.2^, Enforcement of Ordinances ^6.2^ |
|  | Economic Conditions | Economic Circumstances^6.1, 7.5^, Living Wages^12.5^, Material Deprivation^10.6^ |
|  | Structural Conditions | Societal Structure^9.5^, Milieu of Life^10.2^, Education (Quality^6.2, 12.5^, Availability^12.5^), Job Opportunities^12.5^, Security^12.5^, Transport^12.5^, Health Care Services (Quality^9.4^, Availability^10.7, 13.3^, Affordability^10.7, 12.3^, Language Access^12.3^, Insurance Coverage^12.3^, Professional Knowledge^14.12^ and Behaviour^14.18^), Lay Healthcare System^14.14^, Information Media^10.4^ |
|  | **D. Psycho-Social Environment**^14.1^, Personal niche^7.3^ | |
|  | Identity^2.1, 7.1, 14.7^ | Targets, Values, and Norms ^2.6^, Education^10.3^, Employment^10.3^, Socialisation^14.3^, Biography^1.11, 14.4^, Interaction (Caregiver-Child Interaction ^9.1^, Patient-Clinician Relationship^9.4^) |
|  | Personality^15^ | Basic Areas of Personality^7.1^ (Ego Functions, Reality Testing, Self-Esteem, Identity) |
|  | Health Concepts | Health Beliefs^14.9^, Health Awareness^14.10^**,** Specific Health Cognitions^14.11^, Treatment Preferences^9.4^ |
|  | ***Active Individual*** | |
|  | Lifestyle^2.1, 10.2, 11.3, 14.15^ | Diet^6.3, 11.2, 12.2^, Physical Activity^6.3, 12.2^, Daily Routine^11.3^, Hand Washing^12.2^, Daily Actions^14.13^ |
|  | Behavior^2.1, 6.3, 9.2, 11.3^ | Medical Decision-Making^9.4^, Treatment Preferences^9.4^, Health Screening^6.3^, Response to Discrimination^9.3^ |
|  | **E. Individual Conditions** (Person, Subject, Organism)^14.5^ | |
|  | Biology^9.1, 12.1^ | Age^10.1,12.1^, Sex^12.1^, Ethnicity^10.1^, HIV Status^12.1^, Family Microbiome^9.1^ |
|  | Genetics^10.1, 11.1, 12.1^ | Family Biography Genetics^10.1^, Epigenetics^11.1^, Nutrigenomics^11.1^ |
|  | Physiological Needs^2.6^ | Food^2.6^, Oxygen^2.6^, Sleep^2.6^, Sexuality^2.6^, Physical Activity^2.6^ |
|  | Psychological Needs | Needs for Exploration of the Environment and the Self, Self-Realization, Orientation & Security, Ties & Respect)^2.6^, Stimulation^3.1^, Identification^3.1^, Privacy^3.1^ |
|  | **Resources**  = taken from Antonovsky’s Salutogenic Model: Generalized Resistance Resources^1.3^ | |
|  | **A. Natural Resources** | |
|  | Ecological Resources^2.4^ | Clean and Intact Environment^2.4^, Healthy Food^2.4^ |
|  | Natural Sourrounding^5.2^ | Restorative Environment (Parks, Gardens, Lakefronts)^5.2^, Multifunctional Spaces^3.4^, Identification Space and Recreational Space^3.4^ |
|  | **B. Built-Material Resources** | |
|  | Inanimate Objects^7.6^ | Material Resources^1.3, 2.4, 14.6e^ (e.g., Good Housing Conditions)^2.4^, Preventive Architecture^3^, Curative Architekture^3^, Rehabilitative Architecture^3^ |
|  | Infrastructure | Walkability/Cycleability^3.4^, Multifunctional Spaces^3.4^, Identification Spaces^3.4^ |
|  | Land Use | Physical/Built Resources of the Community^6.2,9.5^ (Museums, Libraries)^6.2^, Recreational Spaces^3.4^ |
|  | **C. Socio-Cultural Resources** | |
|  | Social Resources^14.6b^ | Social Support Systems^2.4, 6.3^ and Resources Available Therein^6.3^ (e.g., Social Support^1.3, 6.3^, Family and Community Functioning ^9.2^, Good Relationships with Important Reference Persons ^2.4, 14.6b^, Self-Help Groups^2.4^, Associations^2.4^), Social Reputation^2.4^, Social Integration^6.3^ and Participation^3.4, 6.3^ (Frequency and Type of Use of Multifunctional Spaces)^3.4^, Influence (Existence of Spaces for Experimentation and Appropriation)^3.4^, Characteristics of Social Coherence^3.4^ |
|  | Cultural Resources | Cultural Stability^1.3^, Religious Communities ^1.3, 2.4^, Philosophy^1.3, 14.6b^, Art^1.3^ |
|  | Legal Resources | Legal System^2.4^ |
|  | Economic Resources | Occupational Resources (e.g. Possession of a Training or Workplace, Control over Work, Ergonomic Working Conditions)^2.4^, Work Functioning^9.2^, Sufficient Income^2.4^ |
|  | Structural Resources | Health System^2.4^ (Preventive Health^1.3^, Availability of Health Services^9.4, 11.5^, Medical Care^11.5^, Drugs^11.5^), Education System^2.4^ (School Functioning)^9.2^, Safety-Net Services^9.4^, Safety^6.3^ |
|  | **D. Psycho-Social Resources** | |
|  | Intra-Individual Resources (Psychological) | Knowledge^1.3^, Intelligence^1.3^, Ego-identity^1.3^, Coping Strategies^1.3, 9.2^, (Action^14.6a^)^C^ompetencies^2.2^, (Health Literacy^9.4^, Performance Skills^13.3^), Self-Efficacy Beliefs^2.2^, Sense of Coherence^2.2.^, Appropriation Potential^3.3^, Impulse Control^13.3^ |
|  | Intra-Individual Resources (Non-Psychological) | Physical Resources^13.3^/Fitness^2.2, 14.6c^, Vaccination^9.1^, Education^13.3^, Insurance Coverage^9.4^ |
|  | Inter-Individual^14.6b^ Resources | Ties^1.3^, Committment^1.3^, Social Resources^13.3^, Herd Immunity^9.1^ |
|  | Local-Individual Resources | Local Ties^3.4^ |
|  | ***Active Individual*** | |
|  | ***Behavior*** | Specific Health Behaviour^2.1, 14.17^, Health Action^2.1, 14.16^, Health Screening^6.3^ |
|  | **E. Individual Resources** | |
|  | Genetic resources | Genetic-Constitutional Generalized Resistance Resources^1.3, 14.6c^ |
|  | Psychological Resources | Personality Traits^2.2, 14.6c^, Resilience^13.3^ |
|  | **Stressors**^1.2^  = combined from Antonovsky’s Salutogenic Model (General Resistance Deficits (GRD)^1.2^) and the Vulnerability-Stress Model (Stress Events^13.2^) | |
|  | **A. Natural Stressors** | |
|  | Bio-Chemical Stressors^1.3^ | Environmental Toxins^4.4, 6.3^ (Toxic Substances^4.4,11.4, 12.5^, Lead^6.3^, Particulates^6.3^, Pathogens^4.4^) |
|  | Exogenous Hazards^4.4^ | Hazardous Conditions^4.4^, Degradation of Ecosystems^4.4^ and the Environment (Air and Water Pollution)^11.4^ |
|  | **B. Built-Material Stressors**^14.19^ | |
|  | Inanimate Stressors | Physical Stressors^1.2, 12.5^, Urban Surrounding^5.3^ |
|  | **C. Socio-Cultural Stressors**^13.2,14.19^ | |
|  | Social Stressors | Social Requirements^2.5^, Social Disadvantages^4.4, 6.3^, Social Divide^4.1^, Discrimination^12.5^ (Local Structural Discrimination, Societal Structural Discrimination)^9.3^, Social Deprivation^10.6^, Unequal Education Opportunities^6.1^, Vulnerability Factors of the Social Environment^13.1^ / Requirements (Social Class^13.1^, Education^13.1^, Family^13.1^, Social Networks^13.1^, Professional Socialisation^13.1^, Norms^13.1^, Neighbournhood^6.3^, Workplace^6.3, 2.5^, Housing Conditions^6.3^) |
|  | Historical Stressors | Alienation from the Indigenous Population from their Lands^4.4^ |
|  | Political Stressors | Unequal Distribution of Political Influence^6.1^ |
|  | Economic Stressors | Economic Disadvantages^4.4^, Target Marketing of Harmful Commodities (e.g. Tobacco, Alcohol, Other Licit and Illicit Drugs)^4.4^, Unequal Distribution of Material Wealth and Employment Opportunities^6.1^, Concentrated Poverty^12.5^, Financial Insecurity^6.3^, Material Deprivation^10.8^ |
|  | Structural Stressors | Inadequate or Degrading Health Care^4.4^, Criminality^6.3, 12.5^, Violenent^12.5^, Police Response^6.3^, Social Disorder^12.5^ |
|  | **D. Psycho-Social Stressors** | |
|  | Intra-Individual Vulnerability^13.1^ | Internal Requirements^2.3^, Loss^13.1^, Trauma^13.1, 4.4^ (e.g. Discrimination^4.4, 9.3^ and other Forms of Mental, Physical and Sexual Trauma^4.4^), Socio-Demographic Limited English^9.3^ |
|  | Inter-Individual Stressors | Psycho-Social Stressors^1.2^, Hyperstimulation^3.2^, Fanatism^3.2^, Anonymity^3.2^, Boredom^3.2^, Indifference^3.2^, Alienation^3.2^ |
|  | ***Active Individual*** | |
|  | *Behavior* | Consumption of Alcohol, Cigarettes or Other Drugs^12.2^ |
|  | **E. Individual Stressors**^13.2,14.19^ | |
|  | Vulnerability^13.1^ | Biological^9.1^ and Individual Vulnerability (Age, Sex, Personality, Temperament, Genetic and Neurobiological Factors)^13.1^, Family History Diseases ^12.1^, Carrying the BRCA1 or BRCA2 (Breast Cancer) Gene ^12.1^ |
| II. | **Mutual Interaction** | Cumulative Interplay of Exposition and Resilience^4.5^, Interaction Between Internal and External Requirements and Resources^2^ |
| III. | **Balance** | Opposite of Imbalance (Numeric Code VI), Balance of Influencing Factors^3.3^ |
| IV. | **Positive Life Experiences** | (Consistency, Participation, Underload-Overload Balance)^1.6^, Impact-Orientated Curriculum Vitae^7.4^ |
| V. | **Sense of Coherence** | (Comprehensibility, Manageability, Meaningfulness)^1.4, 14.8^ |
| VI. | **Imbalance** | State of Tension^1.5, 14.20^ Oversaturation/Undersaturation of Needs^3.2, 7.2^, Vulnerability and Resilience^4.5^, Overuse of Focused Attention^5.1^ |
| VII. | **Physical Processing** | Embodiment^4.3^ |
| VIII. | **Mental Processing** | Perception^3.5^, Experience^2.1^, Appraisal^8.1, 14.1^, Coping^8.2, 13.3, 14.2^, Coping Behavior^2.1, 14.21^ |
| IX. | **Successful Management** | Successful Management^1.7^ |
| X. | **Unsuccessful Management** | Unsuccessful Management^1.8^ |
| XI. | **Stress** | Stress^1.9, 14.22^, Affective and Physiological Reaction^8.3^ |
| XII. | **Health Continuum** | Ease (Disease)^1.1, 6.4^ Continuum^14.23^ |
