# Supplementary material for: Salutogenic Environmental Health Model—proposing an integrative and interdisciplinary lens on the genesis of health
Source: Front Public Health. 2024 Oct 17;12:1445181. doi: 10.3389/fpubh.2024.1445181 (PMC11524910; doi:10.3389/fpubh.2024.1445181)
Supplement: Supplementary file 2 [file Table_2.DOCX]

Supplementary Material

*Table 2. Assignment to Individual Models*

| 1. **Salutogenic Model - Aaron Antonovsky (1979, 1987):**    1. The **Ease/Dis-ease-Continuum** is influenced by stressors that affect the status of health in pathogenic, neutral or salutary ways, depending on how the state of tension is handled (Antonovsky, 1979).    2. Potential **Stressors** affect the individual and generate a physical and psychical State of Tension (1.5) 🡪 attempt of Coping (1.7 und 1.8) (subjective assessment of stressors and coping strategies are crucial) (Antonovsky, 1979).    3. Generalized Resistance **Resources** (GRR) are the essential potential for coping with stressors (Antonovsky, 1979):  - roots in societal conditions (1.10) and biographical and family conditions (1.11) - lead to better manageability (1.7) - lead to better life experiences (1.6) 🡪 Sense of Coherence (1.4)   1. **Sense of Coherence** as a key concept of salutogenesis = is the deep conviction of people that their lives can in principle be managed - High Sense of Coherence: better management of stressors and selection of the necessary resources for this purpose (Antonovsky, 1997). - 3 components: Comprehensibility, Manageability, and Meaningfulness (Antonovsky, 1979, 1987).   1. Psychical and physical **State of Tension** generated by confrontation with Stressors (1.5) (Antonovsky, 1979).   2. Positive **Life Experiences** generate a Sense of Coherence (1.4) (Antonovsky, 1979).   3. **Successful Management** of the State of Tension (1.5) 🡪 Sense of Coherence (1.4) and Health (Antonovsky, 1979).   4. **Unsuccessful Management** of the State of Tension (1.5) 🡪 Stress (1.9) (Antonovsky, 1979).   5. **Stress** 🡪 Disease (Antonovsky, 1979)   6. **Sociocultural and Historical Context** are societal conditions that are fundamental to the development of GRR (Antonovsky, 1979).   7. **Biographical and Social Sources of GGRs** are fundamental to the development of GRR (Antonovsky, 1979).  1. **SAR-Model – Peter Becker (2006):**   Central assumption: The state of health depends on how well it manages internal and external requirements with the help of external and internal resources (Becker, 2006).   - 1. **Behavior and Experience of the Individuum:** Coping behavior, emotional behavior, health behavior, need satisfaction, life satisfaction (Becker, 2006).   2. **Internal Mental and Physical Resources** refer to the available means of action or characteristics such as Skills, Competence, Self-Efficacy, Sense of Coherence, Personality Traits and Physical Conditions such as physical fitness (Becker, 2006).   3. **Internal Requirements** are derived from Needs, Targets, Values, and Norms of the person concerned (2.6) (Blümel, 2020).   4. **External Resources** are those in the environment (Blümel, 2020): - Social Resources: e.g., social support systems, good relationships with important reference person, associations, religious communities, self-help groups, social reputation. - Occupational Resources: e.g. possession of an education or job, control over work, ergonomic working conditions. - Material Resources: e.g. sufficient income, good housing conditions. - Societal Resources: e.g., education, health, and legal system. - Ecological Resources: e.g. clean and intact environment, healthy food.   1. **External Requirements** are conditions of the environment, such as professional or social demands, that the individual has to deal with (Blümel, 2020). - Mutual coping of requirements through the use or exchange of resources (Becker, 2006).   1. **Needs, Targets, Values, and Norms** of the concerned 🡪 Internal Requirements (2.3). - The most important human needs (Becker, 2006): - Physiological Needs: e.g., food, oxygen, sleep, sexuality, physical activity. - Needs for exploration of the environment and the self. - Self-Realization, Orientation, and Security - Ties and Respect  1. **PAKARA-Model - Vollmer et al. (2020)**   "Humans do not react to their environment, but acquire it" (Vollmer et al., 2020). This means the individual internalization of the environment 🡪 to promote appropriation, urban architecture must respond to fundamental needs (Vollmer et al., 2020).   - 1. Three **Fundamental Needs**: Stimulation, Identification, Privacy.   „Fundamental needs whose degree of saturation is subject to individual expressions and which can be undersaturated or oversaturated by external factors, which include architecture" (Gruebner et al., 2011; Vollmer et al., 2020).   - Promoting these aspects is health-promoting. - Oversaturation and Undersaturation (3.2) of these aspects can lead to mental and somatic illnesses.   1. **Oversaturation and Undersaturation of Fundamental Needs** = two harmful poles of influence (Vollmer et al., 2020):   Hyperstimulation Fanatism Anonymity  Stimulation Identification Privacy  Boredom Indifference Alienation   - **Hyperstimulation** is the sensory and neurological overload leading to stress and mental illnesses. - Excessive **Identification** can turn into settlement **Fanaticism** (negatively affects cohesion, leads to unhealthy privatization of city management). - Indifference arises when city dwellers cannot identify with their place. - **Excess of Privacy** 🡪 Anonymity, Social Isolation (social emptiness, mutual disinterest in city dwellers)**.** - **Lack of Privacy** (lack of retreat and control, beyond one's own limits and self-determination).   1. **Balance of Influencing Factors** is the prerequisite for the appropriation of urban space 🡪 appropriation potential is prerequisite for psychosocial health (Vollmer et al., 2020).   2. **Implications of a Healthy City** (Vollmer et al., 2020)**:**   **Social:**  7. Characteristics of social cohesion  8. local ties  **Environment:**  1. Walkability/Cyclability  2. Multifunctional space  3. Identification space  4. Recreational areas  **Influence:**  6. Existence of space for experimentation and appropriation  **Participation:**  5. frequency and type of use of multifunctional space   - 1. Fundamental Assumption: Environmental influences in urban space do not affect people directly but via their **Subjective Perceptions** (Vollmer et al., 2020).  1. **EcoSocial Theory – Krieger, Nancy (1994):**    1. **Societal Context** (Social and Historical Context) = “i.e. societal divisions involving property, power, resources, and discrimination, including socioeconomic position, race/ethnicity, Indigenous status, gender, sexuality, disability, nativity, and immigrant status” (Krieger, 2019).    2. **Ecological Context:** Biotic and Abiotic Conditions (Krieger, 2011).    3. “**Embodiment**, referring to how we literally incorporate, biologically, the material and social world in which we live” (Krieger, 2019) (4.1 und 4.2)    4. **“Pathways of Embodiment**, referring to how processes of embodiment are shaped simultaneously by histories of societal arrangements of power and property and by constraints and possibilities of our evolved biology, including gene expression—not just gene frequency” (Krieger, 2019).   🡪 Stressors: see Krieger (2011, S. 215, 223).   - 1. “**Cumulative Interplay** of exposure, susceptibility, and resistance across the life course, referring to the importance of timing and accumulation of and responses to embodied exposures, taking into account individuals’ life-course social and biological development as well as the historical generation into which they have been born” (Krieger, 2019).  1. **ART-Model – Kaplan & Kaplan (1989):**   The ability to concentrate or attention restoration, fatigued by increased demands on directed attention, can be restored by exposure to natural environments (R. Kaplan & Kaplan, 1989; Ohly et al., 2016).   - 1. **Overuse of Directed Attention** (e.g., in an Urban Environment (5.3))🡪 health effects 🡪 Restoration through resting of directed attention by means of sleep, meditation, or **Restorative Environment** (5.2) (S. Kaplan & Berman, 2010).   2. **Restorative Environment** is the Natural Environment (e.g., parks, gardens, and lakefronts), “attracting involuntary attention softly while at the same time limiting the need for directing attention„ (S. Kaplan & Berman, 2010) 🡪 Requirements (S. Kaplan & Berman, 2010; Ohly et al., 2016):  1. Soft Fascination = automatic, effortless attention holding stimuli. 2. The environment must be compatible with our intrinsic motivation (not purpose-driven). 3. The environment must be (or appear to be) large enough to explore it or at least imagine exploring it. 4. There must be the possibility “be away” from everyday stresses.   (S. Kaplan & Berman, 2010; Ohly et al., 2016)   - 1. **Urban Environment** 🡪 Overuse of directed attention (5.1) 🡪 health effects (S. Kaplan & Berman, 2010, p. 48).  1. **Social Determinants of Health – Schulz & Northridge (2004):**   The Social Determinants of Health is "a conceptual framework for environmental health promotion that considers dynamic social processes through which social and environmental inequalities—and associated health disparities—are produced, reproduced, and potentially transformed" (Schulz & Northridge, 2004, p. 445).   - 1. **Fundamental** (Macrolevel) = social and economic inequalities 🡪 influence Intermediate Factors (6.2) (Schulz & Northridge, 2004):  1. Natural Environment: topography, climate, water supply. 2. Macrosocial Factors: historical conditions, political and economic order, legal codes, human rights doctrines, social and cultural institutions, Ideologies (racism, social justice, democracy). 3. Inequalities (distribution of material wealth, employment opportunities, educational opportunities and political influence).    1. **Intermediate** Factors (Meso/Community Level) 🡪 influence Proximate Factors (6.3) (Schulz & Northridge, 2004): 4. Built Environment: Land Use (industrial, residential; mixed use or single use), Transportation Systems, Services (shopping, banking, health care facilities, waste transfer stations), Public Resources (parks, museums, libraries), Zoning Regulations, Buildings (housing, schools, workplaces). 5. Social Context: Community Investment (economic development, maintenance, police services), Policies (public, fiscal, environmental, workplace), Enforcement of Ordinances (public, environmental, workplace), Community Capacity, Civic Participation & Political Influence, Quality of Education.    1. **Proximate** (Micro/Interpersonal Level) 🡪 result in Health & Well-Being (6.4) (Schulz & Northridge, 2004): 6. Stressors: Environmental, Neighborhood, Workplace and Housing Conditions, Violent Crime & Safety, Police Response, Financial Insecurity, Environmental Toxins (lead, particulates), Unfair Treatment. 7. Health Behaviors: Dietary Practices, Physical Activity, Health Screening. 8. Social Integration and Social Support: Social Participation and Integration, Shape of Social Networks and Resources available within networks, Social Support.    1. **Health and well-being** (Schulz & Northridge, 2004): 9. Health Outcomes: Infant and Child Health (low birth weight, lead poisoning), Obesity, Cardiovascular Diseases, Diabetes, Cancers, Injuries and Violence, Respiratory Halth (asthma), Mental Health, All-Cause Mortality. 10. Well-Being: Hope/Despair, Life Satisfaction, Psychological Distress, Happiness, Disability, Body Size and Bdy Image. 11. **The Ecological Circle (Ecological Niche) – Willi (1998):**   The central concept is that human development is shaped by the environment in which they interact, called their personal niche. Psychological well-being is maintained by continuously adapting the environment to fit their personal niche, with the development and differentiation of the niche interacting with personal growth (Willi, 1998; Willi et al., 1999).   - 1. **Fundamental Areas of Personality** are developed through shaping the environment and creating effects (Willi, 1998) 🡪 7.2   2. A persistent **Lack of Response, or a Negative or Distorted Response**, to the four areas of personality is a significant risk to health (Willi, 1998).   3. **Personal Niche** (Willi, 1996) is understood as a person's relational space. It is that part of the social, but also the material environment with which a person actively relates and which they shape themselves (Willi, 1998). According to Willi (2000), the Personal Niche is the place where personal experience is materialized, where one's own history is witnessed and traced. By creating a Personal Niche (home, workplace, business, products of work, own works; Willi, 1998), the person develops and creates an external structure for him/herself (Willi, 2000) 🡪 embedded in the **Environment** (7.5).   4. The **Effect-Guided Life Course** is self-realization, which develops with the Personal Niche in an exciting interaction with the effects set in the Environment (Willi, 1998).   5. "The **surrounding** is the personal world in which an individual lives and to which it feels they belong, without actively influencing it and being personally answered by her. The environment forms the framework and context in which the person unfolds a responsive action" (home country, culture, social class, political and economic circumstances, language, social structure with value system and rules of behavior) (Willi, 1998) 🡪 embedded in the **Environment** (7.6).   6. The Environment, according to Willi (1998), is the totality of the Animate and Inanimate Objects that surround the individual, without the individual making them his or her own. - **External Circumstances** (7.5 and 7.6) can challenge or overwhelm effective niche design and thus personal development (Willi, 2000)**.**  1. **Transactional Model of Stress – Lazarus & Folkman (1987):**    1. **Cognitive Appraisal** 2. **“Primary appraisal** is concerned with the motivational relevance of what is happening, that is, whether something germane to our well-being is involved” (Lazarus & Folkman, 1987, p. 145). 3. “**Secondary appraisal** is a crucial supplement to primary appraisal since harm, threat, challenge, and benefit depend also on how much control we think we can exert over outcomes. If there is a risk of a damaging outcome but one is confident that this can be prevented, threat is apt to be absent or minimal” (Lazarus & Folkman, 1987, p. 146).   **🡪 “**Appraisal concerns the implications of that information for one’s personal well-being” (Lazarus & Folkman, 1987, p. 145). If a situation is appraised as profitable 🡪 no stress arises; if the perceived situational demands strain or overstrain one's own resources 🡪 stress arises (Knoll, 2022).   - 1. **Coping** is the process of managing the discrepancy between perceived demands and resources (Knoll, 2022).  1. **Problem-Oriented Coping** is the troubled person-environment relationship (Lazarus & Folkman, 1987). 2. **Emotion-Focused Coping** managing emotional distress (Lazarus & Folkman, 1987).    1. Criteria: Appraisal and Coping initially influence the immediate affective and physiological reactions to the experienced stress episode (stress reactivity). These in turn have an impact on the long-term emotional, health and social adjustment of the individual (Knoll, 2022). 3. **The National Institute on Minority Health and Health Disparities (NIMHD) Research Framework** (Alvidrez et al., 2019)**:**   is "a tool for conceptualizing and depicting the wide array of determinants that promote or worsen minority health or cause, sustain, or reduce health disparities" (Alvidrez et al., 2019, p. 16).   \|  \| **Individual** \| **Interpersonal** \| **Community** \| **Societal** \| \| --- \| --- \| --- \| --- \| --- \| \| - 1. **Biological** \| Biological Vulnerability and Mechanisms \| Caregiver-Child Interaction  Family Microbiome \| Community Illness Exposure  Herd Immunity \| Sanitation Immunization  Pathogen exposure \| \| - 1. **Behavioral** \| Health Behaviors Coping Strategies \| Family Functioning  School/Work Functioning \| Community Functioning \| Policies and Laws \| \| - 1. **Socio-Cultural Environment** \| Sociodemographic Limited English  Cultural Identity  Response to Discrimination \| Social Networks  Family/Peer Norms  Interpersonal Discrimination \| Community Norms  Local Structural Discrimination \| Societal Norms  Societal Structural Discrimination \| \| - 1. **Health Care System** \| Insurance Coverage  Health Literacy  Treatment Preferences \| Patient-Clinician Relationship  Medical Decision-Making \| Availability of Health Services  Safety Net Services \| Quality of Care Health  Policies \| \| - 1. **Physical/ Built Environment** \| Personal Environment \| Household Environment  School/Work Environment \| Community Environment  Community Resources \| Societal Structure \|   *Tab. I. NIMHD based on Alvidrez et al. (2019, S. 17)*   1. **Eight Classes of Key Factors (Determinants’) that influence health status and quality of life (Lawrence, 2011):**    1. **Family Biography Genetics, Personal Factors** (age, ethnicity, etc.)    2. **Milieu of Life, Lifestyle, Behavior**    3. **Education and Employment**    4. **Information Media**    5. **National** and **Local Policies**    6. **Material** and **Social Deprivation**    7. **Health Services** (availability and affordability)    8. **Qualities of the Environment** are the sum of all conditions under which people live or develop**.** 2. **Four Pillars of Health (Patwardhan et al., 2015):**   "When any one or more of these [four pillars] is compromised, health is at risk” (Patwardhan et al., 2015, p. 63).   - 1. **Genetics** (epigenetics, nutrigenomics) (Patwardhan et al., 2015).   2. **Nutrition** (food, diet, cuisines)🡪 "It has individual, family, and community dimensions" (Patwardhan et al., 2015, p. 63).   3. **Lifestyle** (daily routine, behavior) 🡪 "are totally in our hands, and hence are called modifiable factors" (Patwardhan et al., 2015, p. 62).   4. **Environment** (pollution, epigenetics) 🡪 "In general, urban communities face problems related to environmental degradation, and air and water pollution; rural communities face problems related to sanitation, hygiene, insecticides, pesticides, and agrochemicals" (Patwardhan et al., 2015, p. 64).   5. **Medical Care** (drugs, services) 🡪 support system when one of the 4 pillars is compressed (Patwardhan et al., 2015).  1. **Determinants of Health (ODPHP; Office of Disease Prevention and Health Promotion, 2014):**    1. **Biology and Genetics** (age, sex, HIV status, family history diseases, inherited conditions, carrying the BRCA1 or BRCA2 gene, family history of heart disease).    2. **Individual Behavior** (diet, physical activity, alcohol, cigarette, and other drug use, hand washing).    3. **Health Services** ("Both access [lack of availability, high cost, lack of insurance coverage, limited language access] to health services and the quality of health services can impact health.").    4. **Policymaking** (at the local, state, and federal level e.g., taxes on tobacco).    5. **Social Factors**🡪 conditions of the environment in which people are born, live, learn, play, work, and age: 2. Social Determinants 🡪 Availability of Resources to Meet Daily Needs (educational and job opportunities, living wages); Social Norms and Attitudes (discrimination); Exposure to Crime, Violence, and Social Disorder (concentrated poverty), Quality of Schools, Transportation Options; Public Safety; Residential Segregation 3. Physical Determinants 🡪 Natural Environment (plants, weather, climate change); Built Environment (buildings, transportation), Worksites, Schools, and Recreational Setting; Housing, Homes, and Neighborhoods; Exposure to Toxic Substances and other Physical Hazards; Physical Barriers (especially for people with disabilities) Aesthetic Elements (good lighting, trees, benches). 4. **Vulnerability-Stress-Model (see Roch and Hampel 2019; Wirtz 2022; Wittchen and Hoyer 2011):**   The fundamental assumption of the vulnerability-stress model is that these four components [vulnerability, stress, resilience, coping] only lead to a disease in their interaction (Roch & Hampel, 2019b, p. 250).   - 1. **Vulnerability** is the susceptibility to disease (genetic or acquired) 🡪 it arises from biological, psychological, or social conditions (= contextual conditions) 🡪 it leads to health burdens in connection with Stress Events (14.2) (Roch & Hampel, 2019b, p. 250).  1. Intra-Individual Vulnerability (age, gender, personality, temperament, genetic and neurobiological factors, loss, and trauma). 2. Vulnerability Factors of the Social Environment (social class, education, family, social networks, professional socialization, norms) (Wirtz, 2022).    1. **Stress Events** include any situation that makes biological, psychological or social demands on a person and triggers an adaptive response 🡪 daily hassles (minor stressors) and serious life events (major stressors) (Roch & Hampel, 2019b, p. 250).    2. **Modifying Variables** influence the characteristics of mental disorders' characteristics and their acute and long-term consequences (Wirtz, 2022). 3. **Psychological Factors** as Modifying Variables (resilience, coping, social support, previous disorders, dysfunctional cognitions and schemas, ...)".   **Resilience** is a capacity that enables a favorable response to the situation and a health-promoting coping with the stress despite severe stress **(**and is thus a protective factor) 🡪 can be based on social (social support), socioeconomic (SEP), biological or psychological factors (Roch & Hampel, 2019b, p. 250) (= context conditions).  **Coping** is important for processing the stressful situation (affects **Mental Processing**) 🡪 describes the extent to which stress can be responded to flexibly and efficiently (Roch & Hampel, 2019b, p. 250).   1. **Developmental Factors** as a Modifying Variable (education, impulse control, physical and social factors, performance skills) (Wirtz, 2022). 2. **Integrative Model of Salutogenic (Faltermaier et al., 2017):**    1. **Society, Environment, Life World** 🡪 fundamental for 14.6    2. **Socio-Cultural and Historical Conditions** 🡪 fundamental for 14.6    3. **Socialization** 🡪 fundamental for 14.6    4. **Curriculum Vitae and Biographical Conditions** 🡪 fundamental for 14.6    5. **Person, Subject, Organism**    6. **Health Resources** are a prerequisite for coping with stress and for health-related activities.   They are permanently available strengths/characteristics of a person, social group, environment 🡪 enable or facilitate positive influence on the continuum**.** 🡪 rooted in 14.1; 14.2; 14.3; 14.4   1. **Personal-Psychological** (personality traits, competences). 2. **Social-Interpersonal** (social environment, living environment, social relationships). 3. **Physical-Constitutional** is the constitution of the body and the functioning of the organism (immune competence, vegetative and cardiovascular system, physical fitness, body awareness). 4. **Socio-Cultural** (cultural stability, philosophical convictions). 5. **Physical** (availability of money, assets, goods, services).    1. "Subjective references to health take place in the context of a certain self-perception or personal **identity**, and also have something to do with the goals and priorities a person sets for his or her life" (translated from Faltermaier et al., 1998, quoted from Faltermaier 2017).    2. A **Sense of Coherence** (deep conviction that life is mostly meaningful, understandable and that problems can be overcome) arises from existing resources and positive life experiences 🡪 Promoting Coping Behavior (14.21) and preventing States of Stress (14.22).    3. **Health Beliefs** are subjective health concepts of medical laypersons 🡪 condition for 14.9.    4. **Health Consciousness** encompasses subjective conceptions of one's own health, including cognitive, emotional and motivational aspects in relation to self-perception and the social/material environment, which develop in a biographical and socially harmonious manner**.**    5. **Specific Health Cognitions** are individual beliefs or expectations that are understood as predictors of Health Behavior (14.17).    6. **Professional Knowledge** has an impact on the health of the population through Professional Behavior (14.18) as part of the professional health care system.    7. **Everyday Activities** encompass a person's dominant areas of activity, such as work and household, family, leisure and social relationships, into which Health Activities (14.16) must be integrated.    8. The majority of services to maintain the health of the population and to provide health care are provided by the **Lay Health Care System**. The main actors are individuals, informal social networks (partners, family, friends, relatives, colleagues), self-help initiatives and groups involved in health policy, and municipal institutions.    9. **Lifestyle** influences health behavior (14.16) and is divided into (1) lifestyle without preventive health behavior, (2) lifestyle with specific health behavior and (3) lifestyle with multidimensional or integrated health behavior.    10. **Health Behavior** is a "set of activities that people undertake in their daily lives with the aim of maintaining good health" (p. 232).    11. **Specific Health Behavior** is defined by experts and 'any activity by a person who feels healthy that is intended to prevent disease or to detect it at a stage when it is not yet symptomatic' (p. 246). It includes, for example, avoiding or reducing smoking, heavy alcohol consumption, risky sexual behavior, reducing obesity, physical inactivity, malnutrition and overeating, and increasing healthy eating, physical activity and compliance, as well as taking advantage of screening, early detection and vaccination.    12. **Professional Behavior** is based on Professional Knowledge (14.12) and, as part of the professional health care system, has an impact on the health of the population.    13. **Requirements/**Stressors (psychosocial, physical)/**Risks** (physical, biochemical) "can arise externally from the social and ecological environment, society, culture and people's living environment, but also internally from the person himself (his requirements of himself) or the organism (mutated cancer cells)". 🡪 Link 1.2    14. **State of Tension** (psycho-emotional, physical) 🡪 Link 1.5    15. **Coping Behaviors** (instrumental problem solving, emotional regulation) depending on situation and needs.    16. **State of Stress** (physiological and emotional) 🡪 Link 1.9    17. The **Health Continuum** is a multidimensional (physical-psychological-social) continuum between maximum and minimum health (positive definition of health), well-being, ability to act, complaints, illnesses. 🡪 Link 1.1 6. **Three-Dimensional Personality** (see Gebhard, 2016a)**:**   In contrast to the Two-Dimensional Personality Model, personality structures develop not only through experiences with oneself and others, but also through interactions with the non-human environment 🡪 interactional structures/relationships between humans and 'place'/nature 🡪 our personalities develop through interactions with things (the world becomes familiar through relationships) (Gebhard, 2016b):   - 1. The **Subject** has characteristics derived from the environment and the individual species.   2. Humans feel a sense of **Kinship** with things, landscape and nature. This feeling allows for a central emotional orientation and develops from the unity perceived as a child between humans and elements of the non-human environment. A successful relationship results in a familiarity with the world and non-human objects 🡪 Link 16.6   3. **Object Representations** include physical objects and associated experiences of interacting with them. These mental representations go beyond reproducing the external world and are charged with symbolic meaning. They intensify the aforementioned aspect of the relationship to the objects in an atmospheric way, particularly influencing one's self and having an identity-forming effect.   4. The **Aensory act of Perception** is the symbolic transfer from outside to inside.   5. The **Human Environment** describes personal "objects", i.e. the "human being" as a social being can only be understood against the background of his common history with personal "objects".   6. It is within the **Non-Human Environment** (landscapes, animals, plants, inanimate objects) that the ego and interpersonal relationships develop.   7. A **Place** is a neutral space that becomes a 'place' through interaction in and with it**.**   8. **Self-Objects** are external objects. They are experienced (at least in part) as part of the self and contain emotional meaning. |
| --- | --- | --- | --- | --- | --- | --- | --- | --- | --- | --- | --- | --- | --- | --- | --- | --- | --- | --- | --- | --- | --- | --- | --- | --- | --- | --- | --- | --- | --- | --- |

References

Alvidrez, J., Castille, D., Laude-Sharp, M., Rosario, A., & Tabor, D. (2019). The National Institute on Minority Health and Health Disparities Research Framework. *American Journal of Public Health*, *109*(S1), S16–S20. https://doi.org/10.2105/AJPH.2018.304883

Antonovsky, A. (1979). *Health, stress, and coping* (1st ed.). Jossey-Bass Publishers.

Antonovsky, A. (1987). *Unraveling the mystery of health: How people manage stress and stay well* (1st ed). Jossey-Bass.

Antonovsky, A. (1997). Salutogenese. *Zur Entmystifizierung Der Gesundheit. Tübingen*, *119*.

Becker, P. (2006). *Gesundheit durch Bedürfnisbefriedigung*. Hogrefe.

Blümel, S. (2020). Systemisches Anforderungs-Ressourcen-Modell in der Gesundheitsförderung. *Leitbegriffe Der Gesundheitsförderung Und Prävention: Glossar Zu Konzepten*, Strategien und Methoden. https://doi.org/10.17623/BZGA:224-I121-2.0

Faltermaier, T., Kühnlein, I., & Burda-Viering, M. (1998). *Gesundheit im Alltag: Laienkompetenz in Gesundheitshandeln und Gesundheitsförderung*. Juventa-Verl.

Faltermaier, T., Selg, H., & Ulich, D. (2017). *Gesundheitspsychologie* (B. Leplow & M. von Salisch, Eds.; 2., überarbeitete und erweiterte Auflage). Verlag W. Kohlhammer.

Gebhard, U. (2016a). Zum Zusammenhang von Persönlichkeitsentwicklung und Landschaft. In U. Gebhard & T. Kistemann (Eds.), *Landschaft, Identität und Gesundheit. Zum Konzept der Therapeutischen Landschaften* (pp. 169–184). Springer VS.

Gebhard, U. (2016b). Zum Zusammenhang von Persönlichkeitsentwicklung und Landschaft. In U. Gebhard & T. Kistemann (Eds.), *Landschaft, Identität und Gesundheit. Zum Konzept der Therapeutischen Landschaften* (pp. 169–184). Springer VS.

Gruebner, O., Staffeld, R., Khan, M., Burkart, K., Krämer, A., & Hostert, P. (2011). Urban health in megacities: Extending the framework for developing countries. *Magazine of the International Human Dimensions Programme on Gloabel Environmental Change*, *1*, 40–47.

Kaplan, R., & Kaplan, S. (1989). *The experience of nature: A psychological perspective*. Cambridge University Press.

Kaplan, S., & Berman, M. G. (2010). Directed Attention as a Common Resource for Executive Functioning and Self-Regulation. *Perspectives on Psychological Science*, *5*(1), 43–57. https://doi.org/10.1177/1745691609356784

Knoll, N. (2022). Stressmodell, transaktionales. In M. A. Wirtz (Ed.), *Lexikon der Psychologie*. Dorsch. https://dorsch.hogrefe.com/stichwort/stressmodell-transaktionales

Krieger, N. (1994). Epidemiology and the web of causation: Has anyone seen the spider? *Social Science & Medicine*, *39*(7), 887–903. https://doi.org/10.1016/0277-9536(94)90202-X

Krieger, N. (Ed.). (2011). *Epidemiology and the people’s health*. Oxford University Press. https://doi.org/10.1093/acprof:oso/9780195383874.001.0001

Krieger, N. (2019). A Critical Research Agenda for Social Justice and Public Health: An Ecosocial Proposal. In B. S. Levy (Ed.), *Social Injustice and Public Health* (pp. 531–552). Oxford University Press. https://doi.org/10.1093/oso/9780190914653.003.0026

Lawrence, R. J. (2011). Understanding Environmental Quality Through Quality of Life (QOL) Studies. In J. O. Nriagu (Ed.), *Encyclopedia of Environmental Health* (pp. 518–525). Elsevier. https://doi.org/10.1016/B978-0-444-52272-6.00226-9

Lazarus, R. S., & Folkman, S. (1987). Transactional theory and research on emotions and coping. *European Journal of Personality*, *1*(3), 141–169. https://doi.org/10.1002/per.2410010304

ODPHP (Office of Disease Prevention and Health Promotion. (2014). *Determnants of Health* [Letter to U.S. Department of Health and Human Services]. https://www.healthypeople.gov/2020/about/foundation-health-measures/Determinants-of-Health

Ohly, H., White, M. P., Wheeler, B. W., Bethel, A., Ukoumunne, O. C., Nikolaou, V., & Garside, R. (2016). Attention Restoration Theory: A systematic review of the attention restoration potential of exposure to natural environments. *Journal of Toxicology and Environmental Health, Part B*, *19*(7), 305–343. https://doi.org/10.1080/10937404.2016.1196155

Patwardhan, B., Mutalik, G., & Tillu, G. (2015). Chapter 3—Concepts of Health and Disease. In B. Patwardhan, G. Mutalik, & G. Tillu (Eds.), *Integrative Approaches for Health* (pp. 53–78). Academic Press. https://doi.org/10.1016/B978-0-12-801282-6.00003-6

Roch, S., & Hampel, P. (2019a). Modelle von Gesundheit und Krankheit. In R. Haring (Ed.), *Gesundheitswissenschaften* (pp. 247–256). Springer.

Roch, S., & Hampel, P. (2019b). Modelle von Gesundheit und Krankheit. In R. Haring (Ed.), *Gesundheitswissenschaften* (pp. 247–256). Springer.

Schulz, A., & Northridge, M. E. (2004). Social determinants of health: Implications for environmental health promotion. *Health Education & Behavior : The Official Publication of the Society for Public Health Education*, *31*(4), 455–471. https://doi.org/10.1177/1090198104265598

Vollmer, T. C., Koppen, G., & Kohler, K. (2020). Wie Stadtarchitektur die Gesundheit beeinflusst: Das PAKARA-Modell. *Bundesgesundheitsblatt - Gesundheitsforschung - Gesundheitsschutz*, *63*(8), 972–978. https://doi.org/10.1007/s00103-020-03188-7

Willi, J. (1996). *Ökologische Psychotherapie* (R. Frei, Trans.). Hogrefe Verl. für Psychologie.

Willi, J. (1998). Die ökologische Dimension der Psychotherapie. *Psychotherapeut*, *43*(2), 69–79. https://doi.org/10.1007/s002780050101

Willi, J. (2000). Nische, persönliche. In G. Stumm & A. Pritz (Eds.), *Wörterbuch der Psychotherapie* (pp. 464–465). Springer Vienna. https://doi.org/10.1007/978-3-211-99131-2_1241

Willi, J., Toygar-Zurmühle, A., & Frei, R. (1999). Die Erfassung der persönlichen Nische als Grundlage der supportiven Psychotherapie. *Der Nervenarzt*, *70*(9), 847–854. https://doi.org/10.1007/s001150050523

Wirtz, M. A. (2022). Vulnerabilitäts-Stress-Modell. In *Lexikon der Psychologie*. Dorsch. https://dorsch.hogrefe.com/stichwort/vulnerabilitaets-stress-modell#search=eda873c485fc2c72d940ff4dca8f89a4&offset=0

Wittchen, H.-U., & Hoyer, J. (2011). Was ist klinische Psychologie? Definitionen, Konzepte und Modelle. In H.-U. Wittchen & J. Hoyer (Eds.), *Klinische Psychologie & Psychotherapie* (2., überarbeitete und erweiterte Auflage, pp. 4–26). Springer.
